# Programming ADAR-recruiting hairpin RNA sensor to detect endogenous molecules

**DOI:** 10.1093/nar/gkae1146

**Published:** 2024-12-03

**Authors:** Pei-Pei Qin, Pin-Ru Chen, Liu Tan, Xiaohe Chu, Bang-Ce Ye, Bin-Cheng Yin

**Affiliations:** Institute of Engineering Biology and Health, Collaborative Innovation Center of Yangtze River Delta Region Green Pharmaceuticals, College of Pharmaceutical Sciences, Zhejiang University of Technology, No.18 Chao Wang Road, Gongshu District, Hangzhou 310014, China; Institute of Engineering Biology and Health, Collaborative Innovation Center of Yangtze River Delta Region Green Pharmaceuticals, College of Pharmaceutical Sciences, Zhejiang University of Technology, No.18 Chao Wang Road, Gongshu District, Hangzhou 310014, China; Institute of Engineering Biology and Health, Collaborative Innovation Center of Yangtze River Delta Region Green Pharmaceuticals, College of Pharmaceutical Sciences, Zhejiang University of Technology, No.18 Chao Wang Road, Gongshu District, Hangzhou 310014, China; Institute of Engineering Biology and Health, Collaborative Innovation Center of Yangtze River Delta Region Green Pharmaceuticals, College of Pharmaceutical Sciences, Zhejiang University of Technology, No.18 Chao Wang Road, Gongshu District, Hangzhou 310014, China; Institute of Engineering Biology and Health, Collaborative Innovation Center of Yangtze River Delta Region Green Pharmaceuticals, College of Pharmaceutical Sciences, Zhejiang University of Technology, No.18 Chao Wang Road, Gongshu District, Hangzhou 310014, China; Lab of Biosystem and Microanalysis, State Key Laboratory of Bioreactor Engineering, Shanghai Collaborative Innovation Center for Biomanufacturing Technology, East China University of Science and Technology, No.130 Meilong Road, Xuhui District, Shanghai 200237, China; Institute of Engineering Biology and Health, Collaborative Innovation Center of Yangtze River Delta Region Green Pharmaceuticals, College of Pharmaceutical Sciences, Zhejiang University of Technology, No.18 Chao Wang Road, Gongshu District, Hangzhou 310014, China; Lab of Biosystem and Microanalysis, State Key Laboratory of Bioreactor Engineering, Shanghai Collaborative Innovation Center for Biomanufacturing Technology, East China University of Science and Technology, No.130 Meilong Road, Xuhui District, Shanghai 200237, China; School of Chemistry and Chemical Engineering, Shihezi University, No.221 North Fourth Road, Uighur autonomous region, Shihezi 832000, Xinjiang, China

## Abstract

RNA editing leveraging ADARs (adenosine deaminases acting on RNA) shows promising potential for *in vivo* biosensing beyond gene therapy. However, current ADAR sensors sense only a single target of RNA transcripts, thus limiting their use in different biosensing scenarios. Here, we report a hairpin RNA sensor that exploits new mechanisms to generate intramolecular duplex substrates for efficient ADAR recruitment and editing and apply it to detection of various intracellular molecules, including messenger RNA, small molecules and proteins. We utilize the base pairing interactions between neighbouring bases for enhanced stability, as well as the reverse effects to sense RNA transcripts and single-nucleotide variants with high sensitivity and specificity, irrespective of sequence requirement for complementarity to an UAG stop codon. In addition, we integrate RNA aptamers into the hairpin RNA sensor to realize the detection of the primary energy-supplying molecule, ATP, and a transcription factor, nuclear factor-kappa B (NF-κB), in live cells via a simple conformational change for programming the activation of hairpin RNA. This sensor not only broadens the detection of applicable molecules, but also offers potential for diverse cell manipulation.

## Graphical Abstract

**Graphical Abstract:**
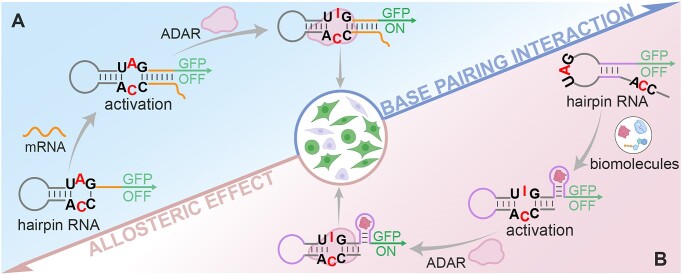

## Introduction

Adenosine deaminases acting on RNA (ADARs) play critical roles in a variety of physiological and pathological processes by regulating gene expression in a post-transcriptional RNA modification process, known as adenosine-to-inosine (A-to-I) RNA editing (1). ADARs act on double-stranded RNA (dsRNA) in a structure-dependent manner and catalyse C6 deamination of A into I (2). This is recognized as guanosine (G) by the cellular machinery due to structural similarity between I and G, enabling the rewriting of genetic information (3,4). This impressive RNA editing feature of ADARs has been creatively exploited for therapeutic applications of the treatment of genetic diseases, such as Duchenne muscular dystrophy (5), Hurler syndrome (6) and Rett syndrome (7), and also holds great potential for the development of RNA sensors in live cells. Most existing ADAR-based sensors, represented by CellREADR (cell access through RNA sensing by endogenous ADAR) (8), RADARS (reprogrammable ADAR sensors) (9) and RADAR (RNA sensing using ADARs) (10), usually deploy a linear, modular RNA molecule containing a sensing sequence reversely complementary to the cellular RNA of interest and an in-frame UAG stop codon as a translation switch for a downstream protein payload. However, the linear design of RNA presents several inherent limitations to using the sensors in various scenarios: (i) high dependence of the mismatch centre sequence in a UAG-CCA pair restricts the selection of the target sequence; (ii) dsRNA substrate format with the assistance of RNA target cannot be extended to the sensing of other endogenous biomolecules; and (iii) dsRNA substrate formed by intermolecular hybridization falls short of achieving the desired sensing efficiency. Moreover, the use of native ADARs with low abundances decreases editing efficiency, leading to limited sensitivity. Therefore, it is highly desirable to provide new insights into the design of ADAR-based sensors with superior performance, universality and applicability.

The factors that affect ADAR editing efficiency are complex, including ADAR enzyme isoforms and expression levels (2,11), substrate dsRNA structure and the neighbouring bases to the editing site (12,13). Therefore, efficient biosensors need to be designed with a combination of factors. Earlier structural and biochemical studies on interactions of human ADAR2 with dsRNA revealed that the deaminase domain of ADAR2 contacts dsRNA crossing a length of ca. 20 base pairs (bp) involving a minor groove near the editing site and two adjacent major grooves (14). We envisioned that disrupting either of the adjacent grooves would affect ADAR2 recruitment, leading to a simple but ingenious strategy to develop a programmable ADAR-recruiting RNA sensor for detection of various endogenous molecules. Inspired by the structure of a naturally occurring hairpin pre-microRNA (pre-miRNA), we engineered a hairpin substrate initially in an inert state, lacking a complete duplex region with a stable editing site. Upon binding of the target molecule, a stable dsRNA containing an intact ADAR binding region is formed. This recruits ADAR to mediate A-to-I editing at the central UAG stop codon, enabling downstream fluorescent protein translation. The sensor realized the detection of arbitrary messenger RNA (mRNA) transcripts of interest, identified single nucleotide mutations and was also extended to detection of small molecules and proteins. We also demonstrated that the active hairpin-structure substrate, in conjunction with an exogenous supply of mutant ADAR2, enabled high editing efficiency.

## Materials and methods

### Reagents and materials

Primers and hairpin RNA sequences used in this study were synthesized by Tsingke Biotechnology Co. Ltd (Beijing, China). Small interfering RNAs (siRNAs) were synthesized by Sangon Biological Co. Ltd (Shanghai, China). Primer, hairpin RNA and siRNA sequences are listed in Supplementary Tables S1–S3. Agarose, bovine serum albumin (BSA) and DH5α chemically competent cells were obtained from Sangon Biological Co. Ltd (Shanghai, China). Dulbecco’s modified Eagle’s medium (DMEM), 10× phosphate-buffered saline (PBS), poly(vinylidene difluoride) (PVDF) membrane, 0.05% Trypsin–ethylenediamine tetraacetic acid (EDTA) and iodoacetic acid (IAA) were purchased from Solarbio Science & Technology Co. Ltd (Beijing, China). Cell Counting Kit-8 (CCK-8), recombinant human IL-1β and Lipo8000 liposomes were purchased from Beyotime Biotechnology Co. Ltd (Nantong, China). Fetal bovine serum (FBS) and Trans 2K Plus II DNA marker were purchased from TransGen Biotech Co. Ltd (Beijing, China). Opti-MEM was obtained from Gibco (Thermo Fisher Scientific, Gaithersburg, USA). Total RNAs extraction kit and cell lysis buffer were purchased from Accurate Biotechnology Co. Ltd (Hunan, China). HyperScript III RT SuperMix for quantitative real-time polymerase chain reaction (qPCR) with gDNA remover and 2× S6 Universal SYBR qPCR Mix were purchased from NOVABIO Biotechnology Co. Ltd (Shanghai, China). 10× DNA loading buffer, 2× Phanta Flash Master Mix, Phanta Max Super-Fidelity DNA polymerase and 2× Rapid Taq Master Mix were purchased from Vazyme Biotechnology Co. Ltd (Nanjing, China). LumiQ universal ECL luminescent liquid was purchased from ShareBio Co. Ltd (Shanghai, China). The antibodies against β-actin and NF-κB were purchased from Abcam (Cambridge, UK). The tips and tubes used were ribonuclease (RNase)-free and did not require RNase inactivation pre-treatment.

The buffers used in this study are the following: (i) 1× sodium dodecyl sulfate (SDS) loading buffer containing 50 mM Tris–HCL, 10% SDS, 30% glycerol, 5% β-mercaptoethanol and 0.02% bromophenol blue; (ii) 1× SDS running buffer containing 25 mM Tris, 192 mM glycerol and 0.10% SDS, pH 8.3; (iii) 1× transfer buffer containing 25 mM Tris and 192 mM glycerol, pH 8.3; and (iv) tris buffered saline with Tween-20 (TBST) buffer containing 15 mM NaCl, 1 mM Tris and 10% Tween 20, pH 8.0.

### Instruments

Polymerase chain reaction (PCR) and qPCR were performed using an Applied Biosystems SimpliAmp PCR thermocycler (Thermo Fisher Scientific, Waltham, MA, USA). The concentrations of plasmid DNA and total RNA were measured by a microplate reader (BioTek Instrument, Winooski, VT, USA) using a Take 3 microplate. Flow cytometry data were obtained using the Beckman cytoFLEX cytometer (Beckman Coulter, Brea, CA, USA). Green fluorescent protein (GFP)-positive cells were screened using the cytoFLEX SRT (Beckman Coulter, Brea, CA, USA).

### Plasmid construction

For reporter plasmid constructs, the sequences of hairpin RNAs (Supplementary Table S2) were overlaid with the coding sequences of blue fluorescent protein (*BFP*) and the *GFP* by PCR using 2× Phanta Flash Master Mix or Phanta Max Super-Fidelity DNA polymerase according to the manufacturer’s instructions. Then, the sequences of *BFP*-hairpin RNA-*GFP* and pUC-cytomegalovirus (CMV) vector were cloned using the ClonExpress Ultra One Step Cloning kit.

The coding sequences for ADAR protein plasmid constructs, including ADAR1^P110^, ADAR1^P150^, ADAR2 as well as two reported variants of ADAR1^P150^ (E1008Q) and ADAR2 (E488Q), were cloned into the pUC-CMV vector, and a flag tag was inserted at the N-terminal end of each gene sequence to facilitate the detection and purification of the expressed proteins.

For plasmid constructs expressing wild-type and mutant genes (Supplementary Table S4), the *NRAS* coding sequence was amplified from complementary DNA (cDNA), and mutations were introduced by PCR. The amplification products were overlapped with ADAR2 (E488Q) coding sequence. Then, ADAR2 (E488Q)-*NRAS* gene was cloned with the pUC-CMV vector, with *NRAS* mRNA transcription driven by a U6 promoter.

### Mammalian cell culture

Cell culture procedures were performed according to methods described in the published literature (15). Briefly, cell lines were cultured in 78.5 cm^2^ cell culture dishes containing 10 ml of DMEM supplemented with 10% FBS at 37°C in a humidified atmosphere containing 5% CO_2_.

### Transfection for plasmids and siRNA

All transfection experiments were conducted by seeding 1 × 10^5^ cells in 24-well plates the day prior to transfection, unless otherwise stated. The growth medium was removed after 24 h and fresh DMEM supplemented with 10% FBS was added prior to transfection. The transfection experiments were performed using Lipo8000 liposomes according to the manufacturer’s instructions.

To optimize the transfection concentration of ADAR2 (E488Q) plasmid, various amounts (50, 100, 150, 200 and 250 ng) of ADAR2 (E488Q) plasmid were transfected into HEK293T cells.

For siRNA transfection, *GAPDH* siRNA, *TP53* siRNA and *HER2* siRNA were used at a final concentration of 100 nM. After 24 h, 400 ng of reporter plasmids and 150 ng of ADAR2 (E488Q) plasmid were co-transfected into HEK293T cells.

For single-nucleotide variant (SNV) detection, 400 ng of SNV sensor plasmid and 150 ng of pUC-CMV-ADAR2 (E488Q)-*NRAS* gene plasmid were co-transfected for exogenous mutation detection. While detecting endogenous mutations, 400 ng of reporter plasmid and 150 ng of pUC-CMV-ADAR2 (E488Q) plasmid were co-transfected.

For ATP detection, HEK293T cells were pretreated with 5 mM Ca^2+^ or 100 μM IAA for 30 min before co-transfecting with 400 ng of ATP sensor plasmid and 150 ng of ADAR2 (E488Q) plasmid.

For NF-κB detection, HEK293T cells were transfected with *NF-κB* siRNA at a final concentration of 100 nM. Subsequently, 400 ng of NF-κB sensor and 150 ng of ADAR2 (E488Q) plasmid were co-transfected into the cells. The following day, cells were stimulated with 50 ng/ml recombinant human IL-1β.

### Flow cytometry analysis

After the cells were transfected and cultured for 48 h, the growth medium was removed from the 24-well plate and washed with 200 μl of PBS. After washing, the cells were digested for 2 min with 200 μl of Trypsin–EDTA (0.05%), and then resuspended in 500 μl of PBS. The resuspended cells were centrifuged at 3000 r.p.m. for 5 min. The supernatant was aspirated and cells were resuspended in 500 μl of PBS. After a second aspiration, the cells were resuspended in 500 μl of PBS and filtered using a 10-μM filter. The cells were analysed using a Beckman flow cytometer, recording 10 000 events per condition at a flow rate of 30 μl/min.

Cells transfected with the pUC-CMV vector served as a gating control. Initial sorting was based on forward/lateral scatter to identify live cell populations, following by sorting based on lateral scatter area/height to distinguish single cells. The BFP signal was used as a fluorescent marker in the cells transfected with reporter plasmid and ADAR2 (E488Q). The normalized fluorescence intensity was determined by the ratio of GFP to BFP fluorescence. To calculate fold activation, the normalized fluorescence intensity in the presence of the target was divided by the normalized intensity in its absence.

### Total RNA extraction, reverse transcription and sequencing

After 48 h transfection, the medium was removed from the 24-well cell culture plate. The GFP-positive cells were sorted and enriched using fluorescence-activated cell sorting (FACS). Total RNA was extracted from 200 μl of cell lysis buffer using a Total RNAs Extraction kit according to the manufacturer’s instructions. The concentration was determined using the microplate reader. The transcription system was prepared according to the manufacturer’s instructions of HyperScript III RT SuperMix, and the cDNA products were generated after incubation at 37°C for 5 min. Then, the cDNA was amplified by PCR using specific primers (Supplementary Table S1) and the resultant PCR products were purified in preparation for Sanger sequencing. For next-generation sequencing (NGS), samples were prepared according to the guidelines provided by Genewiz/Azenta. For each sample, over 50 ng of purified PCR fragment was used for library preparation. The PCR products were treated with End Prep Enzyme Mix for end repairing, 5′ phosphorylation, and dA-tailing in one reaction, followed by T-A ligation to add adaptors to both ends. The adaptor-ligated DNA was purified using DNA Clean Beads. A second PCR reaction was carried out with primers containing sequences that enabled annealing to flow cell for bridge PCR and indexing for multiplexing. The final library products for sequencing were purified by beads, quality checked and sequenced using pair-end 150 bp reads on the illumina Novaseq System.

### RNA editing analysis

Initially, a preliminary statistical analysis was conducted on the raw sequencing data. This was followed by optimization of the sequencing raw data using Cutadapt (version 1.9.1), which involved the removal of the primers and adapter, trimming of the bases with the quality score below 20 at both ends and the exclusion of the reads with an ambiguous *N* base ratio exceeding 10%. Subsequently, statistical analysis of clean data after post-quality control was conducted. Clean reads were merged to generate a complete sequence using Pandaseq software (version 2.7), based on the overlap regions between the Read1 and Read2, and the merged sequences were stored in FASTA (fa) format. Reads were aligned using BWA (v.0.7.18-r1243) and the alignment Binary Alignment/Maps (BAMs) were sorted using Samtools.Finally, RNA editing was analysed using REDitools (v.1.0.3) (16).

### Quantitative real-time PCR assay

The qPCR reaction systems were prepared in a total volume of 20 μl using 2× S6 Universal SYBR qPCR according to the manufacturer’s instructions. The relative transcription levels of genes *GAPDH, TP53, HER2* and *NF-κB* were calculated by normalizing to *β-actin* expression.

### Western blotting experiments

Cells were washed by aspirating growth medium and adding PBS. The washed cells were then diluted with 200 μl of 1× SDS loading buffer and heated at 100°C for 10 min. A gel with 9% sodium dodecyl sulfate–polyacrylamide gel electrophoresis was prepared, and after solidification, 5 μl of cell samples were added into each well. Electrophoresis was conducted at 100 V for about 90 min using 1× SDS running buffer. Proteins were transferred to a PVDF membrane at 200 mA for about 80 min using 1× transfer buffer. The PDVF membrane was blocked with 5% BSA for 90 min and then washed with TBST. Primary antibodies (1:5000 dilution in TBST) were incubated overnight at 4°C, followed by three washes with TBST. Finally, secondary antibodies (1:2000 dilution in TBST) were incubated for 90 min and washed three times with TBST. The results were imaged using LumiQ universal ECL luminescent liquid with a multi-functional fluorescence chemical imaging system (CLINX, Shanghai, China). The complete blots for all prepared proteins, including molecular weight markers, are shown in Supplementary Figure S1.

### Cell viability assay

The day before transfection, 3000 HEK293T cells were seeded into 96-well plates. After 24 h, various concentrations of ADAR2 (E488Q) plasmid were transfected and cultured for 48 h. Subsequently, the medium was replaced with fresh medium supplemented with 10 μl of CCK-8 reagent and incubated for 2 h at 37°C. Absorbance was measured at 450 nm using the microplate reader.

### Statistical analysis

Statistical analyses were performed using GraphPad Prism software version 8.0. All experiments, unless noted otherwise, were conducted with at least three replicates. Data were presented as mean ± s.e.m., with *n* = 3 from three independent experiments. One- or two-way analysis of variance (ANOVA) was used for comparisons of more than two groups. Statistical significance was defined as ****P < 0.0001, ***P < 0.001, **P < 0.01, *P < 0.05 and ns (not significant) for *P* > 0.05.

## Results

### Selection of ADAR protein

**Figure 1.**
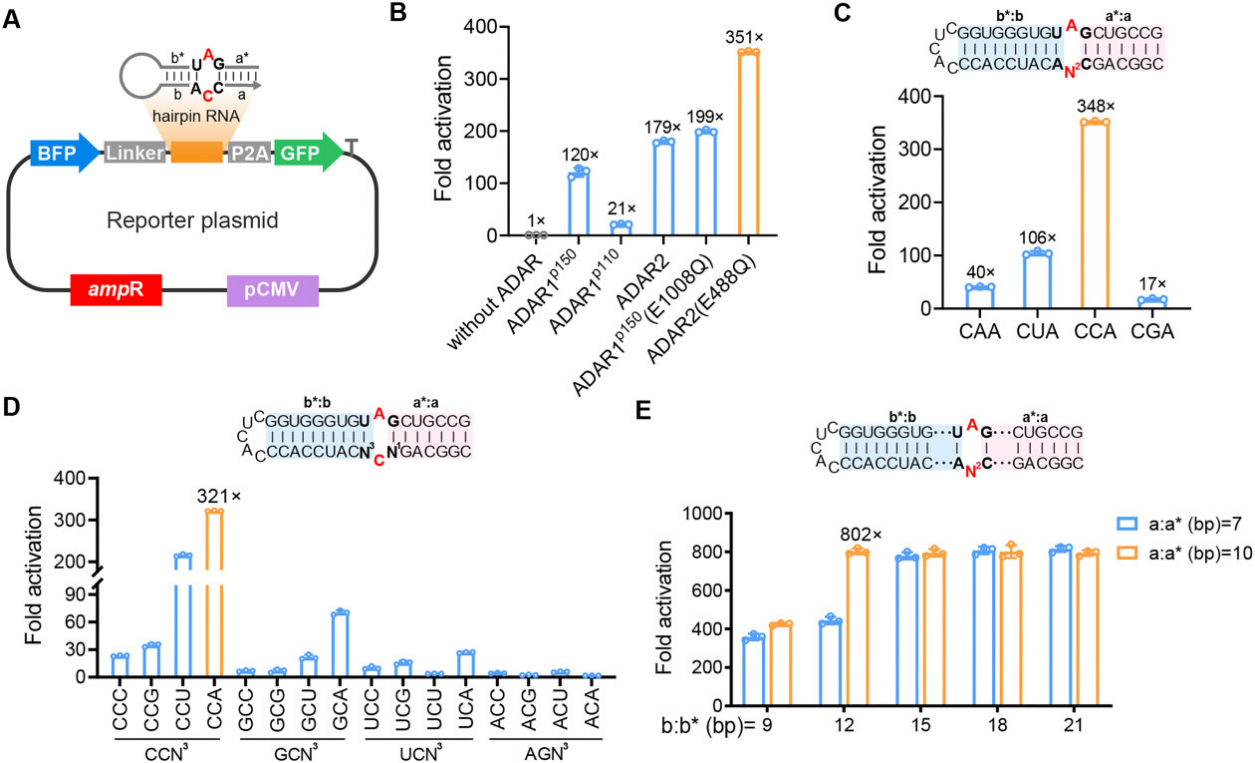
Optimization of ADAR proteins and hairpin RNA substrates. (**A**) Schematic of the reporter plasmid. Note that domains **a**:a* contain a G-C pair and domains **b**:b* contain a U-A pair. (**B**) Fold activation reflects the influence of ADAR on hairpin sensor efficiency upon co-transfection of the reporter and ADAR-coding plasmids. (**C**) Top panel, schematic of hairpin RNA with 5′-CN^2^A-3′ triplet opposite to the UAG. Bottom panel, fold activation reflects the influence of 5′-CN^2^A-3′ triplet on hairpin sensor efficiency. (**D**) Top panel, schematic of hairpin RNA with 5′-N^1^CN^3^-3′ triplet opposite to the UAG. Bottom panel, fold activation reflects the influence of 5′-N^1^CN^3^-3′ triplet on hairpin sensor efficiency. (**E**) Top panel, schematic of the lengths of domains **a**:a* and **b**:b* in hairpin RNA. Bottom panel, fold activation reflects the influence of the lengths of domains **a**:a* and **b**:b* on hairpin sensor efficiency. N denotes A, U, C or G. One-way ANOVA was used for comparison of more than two groups. Values are mean ± s.e.m. with *n* = 3 from three independent experiments.

Prior to fabrication of ADAR-recruiting hairpin RNA sensor, we constructed a reporter plasmid encoding a BFP gene, a hairpin-structured transcript containing a preferred A-C mismatch bubble in a UAG-CCA pair, a GFP gene, a flexible peptide linker (2 × GGGGS) coding sequence and a ribosomal-skipping peptide P2A coding sequence (Figure 1A). Earlier studies demonstrate that the interaction between ADAR2 and dsRNA requires a double-stranded region of ca. 20 bp for substrate recognition and editing (14), and dsRNA unwinding/modifying activity requires at least 15–20 bp (17). Therefore, we adopted a hairpin RNA with a 16-bp intramolecular duplex as a model substrate, in which two complementary regions on either side of the A-C mismatch site are defined as domains **a**:a* (7 bp) and domains **b**:b* (9 bp), respectively (Figure 1A). After transfection of the reporter plasmids to cells, UAG-CCA-containing transcripts recruit ADARs for catalysis of A-to-I transitions, thus altering in-frame UAG stop codons to UIG and initiating the expression of downstream GFP. Note that BFP-positive cells serve as an internal reference to normalize the percentage of GFP-positive cells.

The ADAR family consists of three gene members that encode five proteins isoforms: ADAR1 (ADAR1^p110^ and ADAR1^p150^), ADAR2 (ADAR2a and ADAR2b) and ADAR3 (18). ADAR1 and ADAR2 have deaminase activities, whereas ADAR3 is catalytically inactive (19,20). Given the significant variation in editing effects across different cell lines, we transfected the reporter plasmids into five cell lines (HepG2, HeLa, MCF7, A549 and HEK293T cells), respectively. The normalized fluorescence intensity ratios (GFP/BFP) varied from 0.01 to 0.05 (Supplementary Figure S2), indicating that endogenous ADARs are not suitable for the development of an efficient RNA sensor (21).

Considering the pivotal role of ADAR in our RNA sensor, we speculated that the exogenous ADAR protein supply might enhance the efficiency of RNA editing. To identify the optimal ADAR proteins for RNA editing, we constructed five ADAR protein plasmids encoding ADAR1^P110^, ADAR1^P150^, ADAR2 and two reported variants of ADAR1^P150^ (E1008Q) and ADAR2 (E488Q) (Supplementary Figure S3A), which feature catalytic domain mutations from glutamic acids to uncharged glutamines (22). Western blot experiments confirmed their expression (Supplementary Figure S3B and C). Notably, we observed that HEK293T cells receiving the ADAR2 (E488Q) plasmid and the reporter plasmid exhibited a 351-fold activation in editing compared with cells without the transfection of ADAR plasmid (Figure 1B). This result was consistent with literature reports that the E488Q mutation, located in the catalytic structural domain of ADAR, is more conducive to base flipping than wild-type ADAR (19). Therefore, we chose ADAR2 (E488Q) as the exogenous supply for subsequent studies.

### Characterization and optimization of hairpin RNA

Next, we explored the substrate preference of ADAR2 (E488Q) on hairpin RNA. Previous research by Kuttan and Bass had extensively studied the editing-site specificity of ADAR2, indicating a preferred editing context involving the stop codon UAG (19). On this basis, we investigated the sequence characteristics of a triplet opposite to the UAG. Four bases (A, U, C and G) in the central position of the triplet 5′-CN^2^A-3′ were successively tested. The results showed that the A-C mismatch in the UAG-CCA pair yielded the highest sensing efficiency (Figure 1C). Maintaining the middle cytidine constant, we designed 16 variants by changing the adjacent two bases in the 5′-N^1^CN^3^-3′ triplet. We observed that the 5′-CCN^3^-3′ triplets, particularly when the 5′-neighbouring base was cytidine, exhibited superior sensing effects. Notably, the CCA motif achieved up to a 321-fold activation (Figure 1D).

Inspired by naturally occurring pre-miRNA, we chose the hairpin structure as a substrate for ADAR recruitment. The interaction between ADAR and pre-miRNA has long been a focus of research, with evidence showing that dsRNAs in the stem-loop structure of miRNAs precursor serve as effective ADAR substrates. For instance, Thomas and Beal reported that dsRNA stem length significantly affected ADAR recruitment and RNA editing efficiency (14). To test this effect, we fabricated a series of hairpin RNAs with variable lengths in domains **a**:a* (7 and 10 bp) and domains **b**:b* (9, 12, 15, 18 and 21 bp), respectively. We observed that the fold activation reached a saturation level of 802-fold when the lengths of domains **a**:a* and **b**:b* were 10 and 12 bp, respectively (Figure 1E). Notably, the sensing level of our hairpin RNA design was greatly improved compared with currently reported ADAR sensors (9,10,21). This may be attributed to two reasons: the high abundances of exogenous ADAR2 (E488Q) supply and the structural advantage of the intramolecular RNA duplex. The first reason was substantiated by data presented in Supplementary Figure S4, which illustrates the impact of exogenous ADAR supplementation on hairpin sensor efficiency. To validate the second factor, we compared our hairpin RNA of an intramolecular duplex (termed Reporter-1) with the reported RNA sensing design of an intermolecular duplex (23) (termed Reporter-2) in sensing efficiency using exogenous ADAR2 (E488Q) (Supplementary Figure S5A). Despite both RNA substrates having identical double-stranded lengths of 23 bp, fluorescence micrographs and flow cytometry results showed that Reporter-1 significantly outperformed Reporter-2, achieving a fold activation difference of 782-fold versus 117-fold (Supplementary Figure S5B). We reasoned that the intermolecular dsRNA substrate may require a longer duplex length for effective RNA editing. For instance, in the LEAPER system, the EGFP-positive cell rate was ca. 60% when using an optimized ADAR-recruiting RNA (arRNA) of 71 nt (23). Although the circular arRNA in the updated LEAPER 2.0 exhibited an improved editing efficiency and specificity (6), its cellular processing was more complex, requiring an extra addition of exogenous nuclease (24). Finally, to minimize the potential damage to cellular transcripts caused by the introduction of exogenous ADAR2 (E488Q), we optimized the transfection concentration of the ADAR2 (E488Q) plasmid and found that cell viability remained unaffected at a plasmid dosage of 150 ng (Supplementary Figure S6), a level we chose for subsequent studies.

### Programming hairpin RNA for endogenous RNA sensing

**Figure 2.**
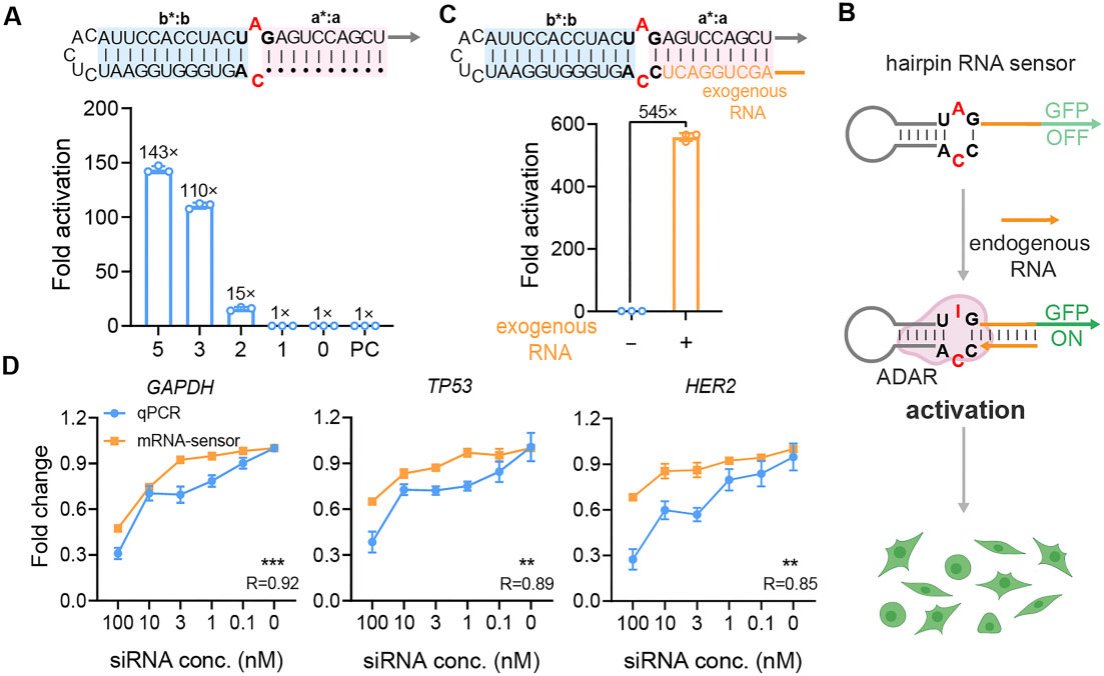
Programming hairpin RNA structure for RNA sensing. (**A**) Top panel, schematic of hairpin RNA with shortened domain **a**. Bottom panel, the effect of shortened domain **a** on hairpin sensor efficiency. PC, positive control with a hairpin RNA deleting domain **a** and a neighbouring cytosine. (**B**) Schematic of the working principle of endogenous RNA detection based on base pairing effects. (**C**) Top panel, schematic of shortened hairpin RNA upon the introduction of exogenous RNA. Bottom panel, the effect of exogenous RNA supply on recovery of hairpin RNA substrate activity. (**D**) Correlation between the knockdown effects of siRNAs with different concentrations and sensing efficiencies by the hairpin RNA sensor targeting *GAPDH*, *TP53* and *HER2* mRNAs. R value denotes Pearson’s correlation between qPCR and the hairpin RNA sensor. For hairpin RNA sensors responding to different RNAs, fold change was normalized to the maximal sensor activation. ***P < 0.001; **P < 0.01. Values are mean ± s.e.m. with *n* = 3 from three independent experiments.

After experimentally confirming the superior sensing performance of hairpin RNA compared with the conventional linear counterparts, we explored the possibility of developing a versatile RNA sensor by manipulating the duplex region of a hairpin RNA through its destruction and reconstruction. We adopted an easy way to disable the RNA duplex by shortening the length of domain **a** but maintaining the length of domains **b**:b* (12 bp) (Supplementary Figure S7). As shown in Figure 2A, we observed a decrease in fold activation to 143-fold when the length of domain **a** was reduced to 5 nt, and a further reduction to near baseline (1-fold) when domain **a** was shortened to a single cytosine base or completely deleted. This result was consistent with the positive control, which involved completely deleting domain **a** and a mismatch cytosine opposite the adenine in the UAG stop codon. These results further confirmed the critical length requirements of the RNA duplex flanking the UAG-CCA pair, suggesting that the reduced ADAR activity could be due to insufficient binding at essential amino acid sites within the ADAR2 deaminase domain (14).

We next attempted to exploit the above hairpin RNA structure with a single cytosine in domain **a** as a sensor module for endogenous RNA sensing. This hairpin structure was selected for two reasons: (i) it exhibited minimal editing activity, providing a near-zero fluorescence background essential for high sensitivity and (ii) the presence of a single cytosine facilitated a complete CCA triplet to pair with UAG, potentially reducing structural strain on the central A-C mismatch and thus enhancing the stability of the editing loop to a certain extent. We hypothesized that the recovery of the stable duplex region within this inert hairpin RNA could be achieved by hybridizing target RNA at the location of domain **a**, utilizing base pairing interactions (Figure 2B). To preliminarily validate this concept, we introduced a plasmid encoding an exogenous RNA, resulting in a fold activation up to 545-fold (Figure 2C). Flow cytometry analysis of the ratio of GFP to BFP signal in BFP and GFP double-positive cells showed 79.85% of the cells with high GFP/BFP ratio in the presence of exogenous RNA, whereas in the absence of exogenous RNA, the GFP/BFP ratio was 0.85% (Supplementary Figure S8). These results indicated that base pairing-aided hybridization between the hairpin sensor and target RNA significantly restored the activity of a stable dsRNA substrate, enabling efficient ADAR recruitment.

To further investigate the influence of base pairing on the hairpin RNA sensor, we conducted a comprehensive analysis of all potential base pair combinations flanking the CG closure of the editing loop. We designed 16 distinct base pair sets (N^4^:N^5^, where N = A, U, G and C) (Supplementary Figure S9A). The results showed that the hairpin RNA sensors were strongly activated when the bases were correctly paired, particularly with U:A and C:G pairs showing 250-fold activation (Supplementary Figure S9A). Meanwhile, we found that the activation fold was greater for U:A than its reverse A:U when N^4^ was A and N^5^ was U. Similarly, the activation fold was higher for C:G than its reverse G:C when N^4^ was G and N^5^ was C. These disparities could be attributed to the distinct stacking effects of the G:C base pairs adjacent to N^4^:N^5^ within the sensor (25,26). Considering potential interferences from RNAs of different species in ADAR recognition, we assessed eight base pair combinations (with N^6^ being either G or C) for sensing efficiency. Supplementary Figure S9B shows that the CCA motif was the most effective for RNA hybridization across species. Subsequently, we explored the impact of varying mRNA lengths on sensor binding, testing target complementary sequence lengths of 9, 18, 27, 39 and 48 nt (Supplementary Figure S10A). We found that a 39 nt length was optimal (Supplementary Figure S10B), which was adopted in our subsequent studies. Furthermore, to confirm the result of A-to-G base editing, we enriched GFP-positive cells co-transfected with the hairpin RNA sensor and ADAR2 (E488Q)-exogenous RNA plasmid via FACS. We extracted genomic RNA of HEK293T cells, performing reverse transcription (RT)-PCR followed by Sanger sequencing and NGS. The sequencing results showed that the stop codon TAG was converted to the tryptophan codon TGG (Supplementary Figure S11A), and the A-to-I (G) conversion rate was about 60% (Supplementary Figure S11B). Additionally, we assessed bystander editing by measuring exogenous RNA regions hybridized to the hairpin RNA sensor by NGS. Supplementary Figure S11C indicates that the highest bystander editing levels were below 2%, confirming minimal off-target effects.

To evaluate the sensitivity of the hairpin RNA sensor, we detected endogenous RNAs with varying expression levels regulated by siRNAs. We used *GAPDH*, *TP53* and *HER2* mRNAs in HEK293T cells as models. For each mRNA transcript, three siRNAs were designed and tested, with the most efficient sequence selected: 71.66% for *GAPDH* mRNA, 63.13% for *TP53* mRNA and 66.44% for *HER2* mRNA (Supplementary Figure S12A–C). We designed three to five potential binding sites for each target, identifying the most effective site for each, and created the corresponding mRNA sensors (Supplementary Figure S12D). These sensors were then used to detect the target mRNAs in HEK293T cells, with the selected siRNAs delivered at concentrations ranging from 0 to 100 nM. qPCR analysis revealed that, despite varying degrees of knockdown, our hairpin sensors consistently displayed a high Pearson correlation with the presence of target RNAs, even at lower levels of *HER2* gene expression, showing a sensitive signalling response (Figure 2D). Furthermore, we tested the A-to-I (G) conversion rate of hairpin RNA sensor in the absence of siRNA interference by NGS. Due to the differences in transcript abundance, the sequencing results showed A-to-I (G) conversion rates of approximately 40%, 10% and 6% for the GAPDH sensor, TP53 sensor and HER2 sensor, respectively (Supplementary Figure S12E). We also tested the specificity of the hairpin RNA sensor. The results showed that the sensor reliably responded to *TP53* mRNA, even in the presence of siRNA targeting *GAPDH* mRNA (Supplementary Figure S12F). Collectively, these findings demonstrated that our designed hairpin RNA sensor can sensitively and selectively detect endogenous mRNA in live cells by leveraging the base pairing effect.

### Sensing SNVs of endogenous RNA

**Figure 3.**
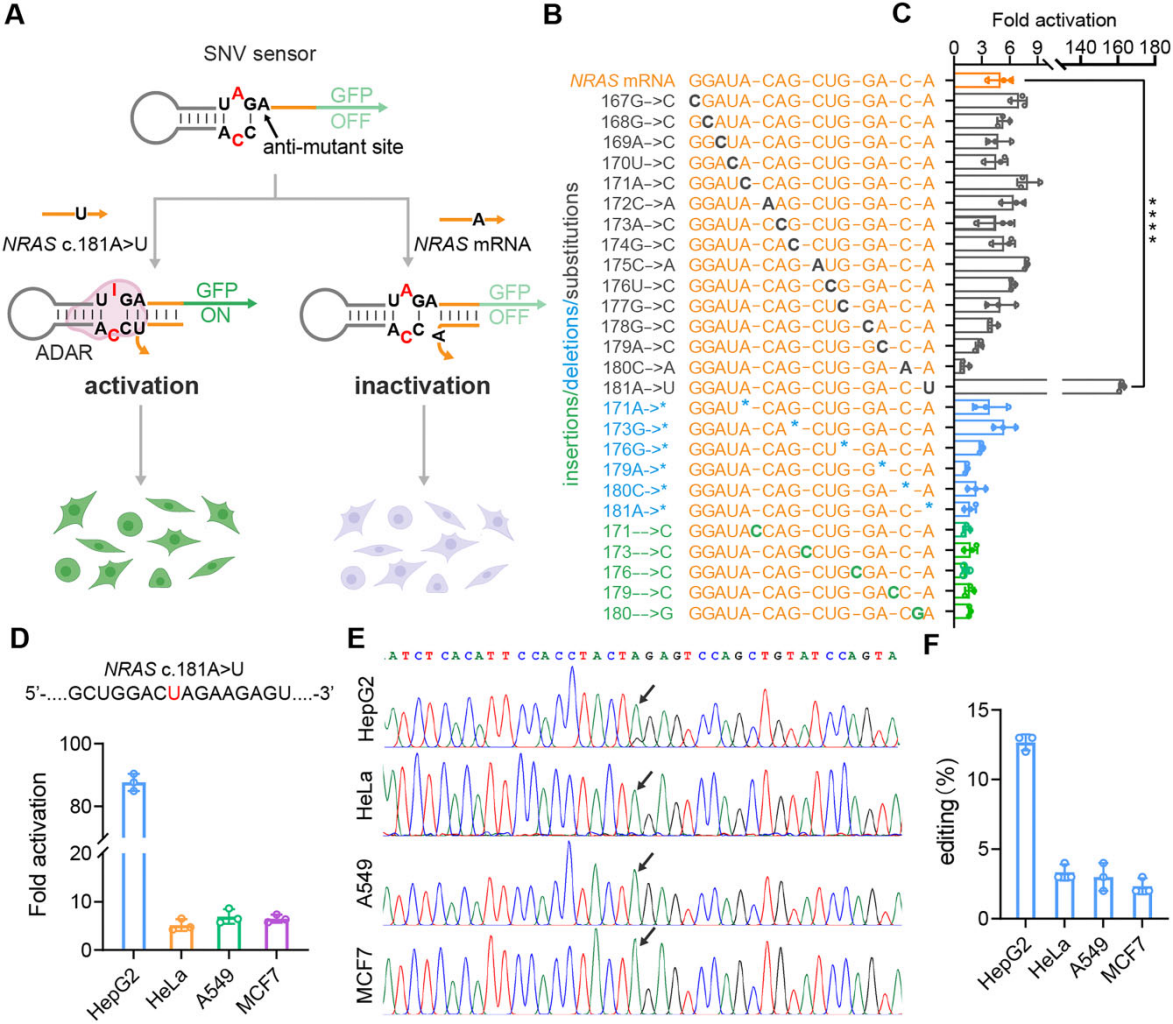
Programming hairpin RNA for detection of SNVs. (**A**) Schematic of the working principle of sensing a single-nucleotide mutation by rationally designing a mutation detection site. (**B**) Sequences of the *NRAS* mRNA and mutant mRNAs (c. 181A > U) with single-nucleotide substitutions, deletions and insertions. (**C**) Sensing specificity of our sensor by measuring fold activation. (**D**) Co-transfections of plasmids encoding SNV sensors and ADAR2 (E488Q) were performed in four cell lines (HepG2, HeLa, A549 and MCF7 cells) for detecting the single-base mutation of *NRAS* mRNA (c.181A > U). (**E**) Sanger sequencing and (**F**) quantification results of the A-to-I editing rate for SNV sensors in the tested four cell lines by NGS. One-way ANOVA was used for comparison of more than two groups. ****P< 0.0001. Values are mean ± s.e.m. with *n* = 3 from three independent experiments.

Base pairing at the nicks provides an additional stability and hybridization efficiency. However, base mismatches could greatly weaken hybridization chain interactions (27). Inspired by this intolerance to mismatches, we intended to program the hairpin RNA structure for sensing SNVs, which are valuable biomarkers for determining protein dysfunction and assessing disease risk (28). As shown in Figure 3A, we used the c.181A > U mutation in the *NRAS* gene as a model. We strategically positioned the mutation detection site adjacent to the 5′-CCA-3′ triplet, slightly modifying the inert hairpin RNA to include an antimutant site A adjacent to the 3′-neighbouring G of the 5′-UAG-3′ triplet. When the *NRAS* c.181A > U mRNA presents a perfectly matched U to A, the hairpin sensor activates, leading to efficient editing on the UAG and downstream GFP expression. Conversely, the wild-type *NRAS* mRNA with a mismatch base, forms a duplex region with two mismatches (A-C and A-A), which are thermodynamically unfavourable ADAR editing.

To investigate the effect of types and positions of single-base mutations on specificity of the engineered hairpin RNA, we designed three sets of RNA targets having three types of single-base mutations: substitutions (grey), deletions (blue), and insertions (green) (Figure 3B). As expected, the closer the mutation site to the UAG-CCA triplet, the higher the specificity was achieved to detect the single nucleotide changes (Figure 3C). Then, we tested other mutations, such as A > C and A > G, and found that significant fluorescence signal was observed only when the bases formed an A-U pair in our SNV sensor for *NRAS* mRNA (c.181A > U) (Supplementary Figure S13). We also modelled mRNA mutation c.181C > G and tested the C-G complementary pairing along with its mutations (C > A, C > U, and C > C). The results showed that precise C-G pairing led to a 160-fold activation, indicating the high specificity of our hairpin RNA sensor for single nucleotide mutation detection. In addition, we found that single-base deletions and insertions in mRNAs did not produce significant responses from the SNV sensor (Figure 3C). We further co-transfected the SNV sensor along with the plasmid encoding the ADAR2 (E488Q) into four cell lines. The results demonstrated that our sensor was able to specifically identify a single-base mutation in endogenous *NRAS* mRNA (c.181A > U) in HepG2 cells (mutant-type heterozygote) among other tested cell lines of wild-type homozygotes (HeLa, A549, and MCF7 cells), with the fold activation up to 87-fold (Figure 3D). Sanger sequencing of the target region showed A/G overlapping peaks at the adenosine site in the TAG, with an editing rate of 13% in HEPG2 cells (Figure 3E and 3F).

### Intracellular ATP detection

**Figure 4.**
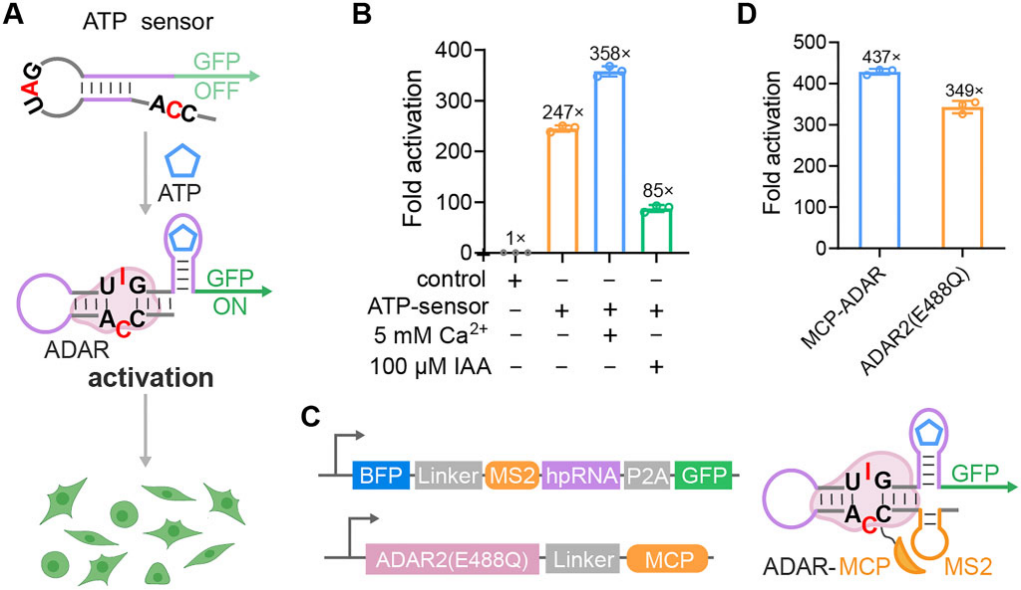
Programming hairpin RNA for intracellular ATP detection. (**A**) Schematic of the principle of ATP detection. (**B**) Effects of exogenous stimulation with IAA and Ca^2+^ on the ATP sensing efficacy. Control group used random sequences instead of ATP aptamer sequences. (**C**) Schematic of plasmid construction and binding principle of MCP-MS2 system in our sensor. (**D**) Comparison of sensing performance of ATP sensor coupled with and without MS2-MCP system. One-way ANOVA was used for comparison of more than two groups. Values are mean ± s.e.m. with *n* = 3 from three independent experiments.

Most ADAR-based sensors exploit an RNA trigger to control gene expression. However, no method has been reported using intracellular biomolecules other than RNA molecules, such as small molecules or proteins, to initiate RNA editing. To overcome this limitation, we introduced an RNA aptamer to establish a linker between target molecules and signalling proteins. We first programmed the hairpin RNA to respond to intracellular ATP, the primary energy currency in living cells (29,30). On the basis of our hairpin RNA with domains **a**:a* and **b**:b* of 10 and 12 bp, we incorporated a 40-nt ATP RNA aptamer (Supplementary Table S5) and its partially complementary sequence (18 nt) to construct a new stem-loop structure, in which the UAG-CCA pair is separated and respectively located at the loop and 5′-overhang end (Supplementary Figure S14A). We predicted the secondary structure of the full-length ATP sensors (including the downstream GFP sequence) and the sensor without ATP aptamer using the RNAfold web server (Supplementary Table S14B and S14C). In the presence of ATP molecules, the binding between aptamer (depicted by a purple line) and ATP disturbs the intramolecular RNA hybridization and enables the re-formation of the dsRNA structure with the UAG-CCA pair, leading to the restoration of editing activity and subsequent expression of downstream signal proteins (Figure 4A).

We tested this design by co-transfecting plasmids encoding ATP sensors and ADAR2 (E488Q) into HEK293T cells. The ATP sensor showed a 247-fold activation compared with the control with a random sequence replacing the ATP aptamer (Figure 4B). To verify the signals originated from ATP, we regulated the intracellular ATP levels by use of calcium ions (Ca^2+^) and IAA, known ATP inducers and inhibitors, respectively (31). After verifying that neither Ca^2+^ nor IAA affected the expression of ADAR2 (E488Q) or the function of the hairpin RNA sensor (Supplementary Figures S15A and S15B), we observed a significant activation increase to 358-fold in cells with 5 mM Ca^2+^, and a noticeable decrease to 85-fold in IAA-treated cells upon the transfection of ATP sensors (Figure 4B). Notably, we observed a relatively lower signal in ATP detection compared with endogenous RNA detection, likely due differences in aptamer-ATP binding affinity versus base-pairing interactions. To enhance ATP sensor performance, we aimed to improve the recruitment of ADAR to the sensor's edit sites by introducing the MS2-MCP system, which leverages the high-affinity interaction between the MS2 bacteriophage coat protein (MCP) and its cognate RNA hairpin aptamer (MS2) (22). We added the MS2 sequence to the 5′ end of the hairpin RNA and linked the gene encoding the MCP protein to the ADAR coding sequence during plasmid construction (Figure 4C). This modification significantly increased the affinity of the ADAR for the hairpin RNA substrate, leading to a higher sensor activation of 437-fold (Figure 4D). We also observed that the MS2-ATP sensor showed increased activation in the presence of Ca^2+^, and despite IAA reducing cell viability, the sensor maintained a higher activation of 245-fold (Supplementary Figure S15C). We speculated that the MCP/MS2 system increased the sensitivity of the ATP sensor, but also potentially due to the increased background editing. To further optimize the sensor, we tested two ADAR2 (E488Q) and MCP fusions, each with a nuclear export sequence (NES) or a nuclear localization sequence (NLS). We observed that the inclusion of the NES further improved ATP sensor performance, yielding a 484-fold activation (Supplementary Figure S16). Taken together these results demonstrated that our hairpin RNA sensor can sensitively detect small molecules in live cells.

### NF-κB detection

**Figure 5.**
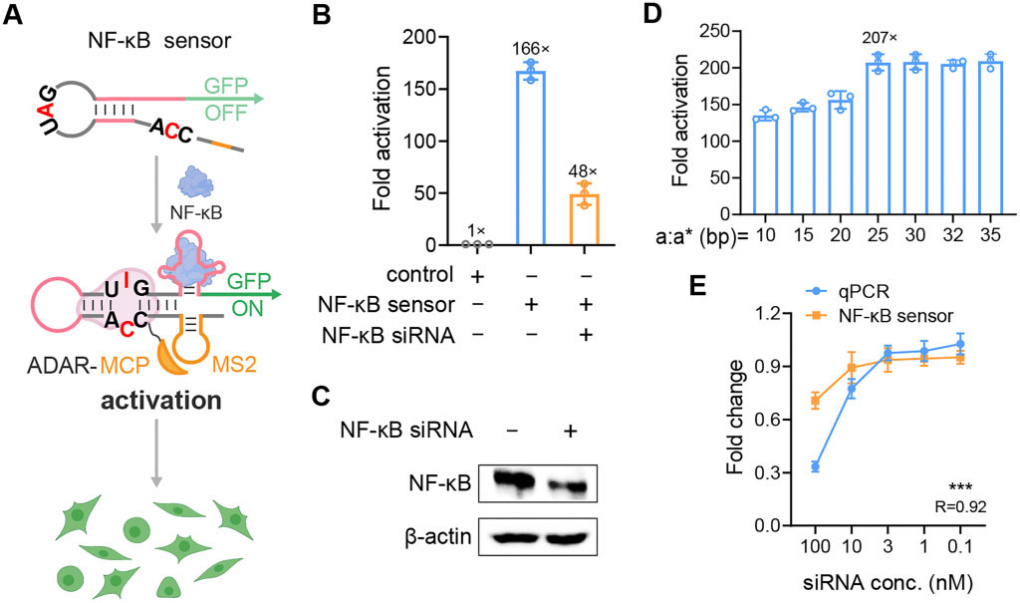
Programming hairpin RNA for intracellular NF-κB detection. (**A**) Schematic of the principle of NF-κB detection using NF-κB RNA aptamer. (**B**) NF-κB sensing in HEK293T cells with different treatments. Control group used random sequences instead of NF-κB aptamer sequences. (**C**) Validation of NF-κB protein knockdown by western blotting. (**D**) Optimization of the length of the domains **a**:a*. (**E**) Comparison of NF-κB protein levels determined by qPCR and fold activation measured by NF-κB sensors in siRNA-treated HEK293T cells. R value denotes Pearson’s correlation between qPCR and the NF-κB sensor. For NF-κB sensor, fold change was normalized to the maximal sensor activation. One-way ANOVA was used for comparison of more than two groups. ***P < 0.001. Values are mean ± s.e.m. with *n* = 3 from three independent experiments.

To further expand the applicable target of our design, we chose a crucial nuclear transcription factor, NF-κB, as a protein model. NF-κB plays a significant role in inflammatory and immune responses (32), and its pathway is closely associated with various human diseases (33,34). For the NF-κB sensor construction, we incorporated a 29-nt NF-κB RNA aptamer with a K_d_ value of 5 nM (Supplementary Table S5) (35) and its partial complementary sequence (18 nt) to form a new stem-loop structure (Supplementary Figure S17A). We used the RNAfold web server to predict both the full-length NF-κB sensors (including downstream GFP sequences) and the sensor lacking NF-κB aptamer (Supplementary Figure S17B and C). As shown in Figure 5A, the RNA aptamer sequence is blocked by its partial complementary sequence, causing the UAG-CCA base pair to be separated. Upon binding of NF-κB to the RNA aptamer, a conformational change occurs in the hairpin RNA, exposing the UAG-CCA base pair. This exposure facilitates the recruitment of ADAR for A-to-I base editing. Consequently, this editing process enables downstream expression of fluorescent proteins, allowing for NF-κB detection. We co-transfected plasmids encoding the NF-κB sensor and ADAR2 (E488Q) into HEK293T cells under external stimulation with 50 ng/ml recombinant human IL-1β. The NF-κB sensor showed a 166-fold activation compared with controls with random sequences replacing the NF-κB aptamer (Figure 5B). We then identified an siRNA sequence that knocked down *NF-κB* mRNA by 61.23% (Supplementary Figure S18) and confirmed its knockdown effect on NF-κB protein levels by western blotting (Figure 5C). Upon pretreatment of HEK293T cells with this siRNA, the fold activation significantly decreased to 48-fold (Figure 5B), validating the robustness of NF-κB sensor.

We observed that the sensing efficiency of NF-κB sensor was significantly lower compared with the ATP sensor and speculated that the short length of 10-bp domain **a**:a* might cause asteric hindrance between NF-κB and ADAR upon NF-κB binding to the RNA aptamer. To confirm this hypothesis, we designed a series of domains **a**:a* with lengths ranging from 10 to 35 bp. As shown in Figure 5D, fold activation gradually increased with the lengths of domains **a**:a*, reaching 200-fold activation with the 25-bp domain **a**:a*. This suggested that a larger spatial configuration could significantly enhance sensor performance. Additionally, we investigated the effect of NLS and NES on NF-κB detection. We observed that incorporating NLS did not significantly improve the performance of the NF-κB sensor compared with the ADAR variant lacking these sequences (Supplementary Figure S19). Previous studies have demonstrated that ADAR2 is predominantly localized in the nucleus (3). Given that the performance of NF-κB sensor was already optimized in the nucleus, the addition of NLS did not further contribute its detection capabilities. Finally, we evaluated our sensor’s ability to detect HEK293T cells with varying NF-κB expression levels, regulated by exogenous delivery of siRNAs (ranging from 0 to 100 nM). As expected, our sensor showed a high Pearson correlation with the qPCR results (Figure 5E). These results suggested that our design can be easily tailored to respond to different biomolecules by changing the RNA aptamers.

## Discussion

In this study, we reported the first example of a programmable ADAR-recruiting hairpin RNA sensor capable of sensitive detection of various endogenous molecules. Notably, we demonstrated that an inactive hairpin RNA can be facilely programmed in response to mRNA via the base pairing effects, allowing for the accurate identification of a single base change with a significant 162-fold activation compared with wild-type RNA. Simultaneously, the proposed hairpin RNA sensors, which leveraged RNA aptamer-mediated conformational changes, were equally sensitive to endogenous molecules such as ATP and NF-κB. Our sensor exhibits several advantageous features for live-cell biomolecule detection over the current ADAR-based RNA sensors.

In recent years, the RNA editing ability of ADAR to precisely edit A to G has shown great promise in gene therapy and is increasingly applied in the biosensing field. The use of linear RNA molecules as sensing elements has bestowed ADAR with multiple applications, including determining cell types, tracking transcriptional states and inducing cell death (9,10). However, the editing efficiency of ADAR is compromised when utilizing linear RNA substrate, constraining the range of detectable molecules. To Address this, we designed hairpin-structured RNAs to enhance editing efficiency by systematically exploring the substrate-selective preferences of ADAR2 (E488Q). We identified a short hairpin RNA with an intramolecular duplex that demonstrated superior editing efficiency compared with the longer dsRNA formed through intermolecular hybridization. It has been demonstrated that many natural RNA substrates have higher selectivity and editing efficiency (36,37). Inspired by pre-miRNAs, our hairpin structure closely resembled these natural RNA substrates, ensuring efficient editing with a design that included 10 and 12 bp flanking the A-C mismatch site, totaling 23 bp (Figure 1E).

Our work introduced a simple yet efficient base pairing interaction to reconstruct active hairpin RNA, overcoming sequence restrictions and enabling the targeting of RNA transcripts without complementarity to the termination codon. Our study demonstrated that UAG-ACC triplex pairing achieved an optimal level of ADAR editing, highlighting the importance of considering ACC sequences in target RNA transcripts. Despite the prevalence of ACC sequences in many transcripts, effective target selection still faces certain limitations. For example, when detecting single nucleotide mutations, only mutations of the type N (U, A and G) > C were feasible. Expanding detection to a broader range of mutation types, including base deletions and insertions, presents additional challenges. However, our hairpin RNA sensor, utilizing base pairing, adeptly addresses these issues. Furthermore, conventional RNA sensors often produce nonsense proteins during translation, which do not participate in the signalling of the sensor or the detection of target molecules. These nonsense proteins would negatively impact sensor performance in living cells and disrupt protein homeostasis. Therefore, it is crucial to design and optimize sensing elements accurately to minimize the production of these unwanted proteins. ADAR-based RNA sensors maintain component invariance when targeting different RNA molecules and are more compact than previous RNA sensors, facilitating their efficient operation in living cells.

In our study, we exogenously expressed mutant ADAR2 to enhance the sensing efficiency. However, overexpressing ADAR2 (E488Q) led to significant off-target editing, with MCP-ADAR2 (E488Q) resulting in 3500 off-target sites (9). This poses potential risks to host cells and limits applications in cell and gene therapy. Given these challenges, future research will focus on improving the editing efficiency using endogenous ADAR. To date, several studies have successfully enhanced the editing efficiency though guide RNA modification (13,23), circular RNA design (6,24) and feedback system development (21). Moving forward, it will be essential to explore additional strategies. For instance, employing signal amplification strategies could convert the lower editing activities into more robust signal outputs, thereby enhancing the sensing efficiency. Time-controlled expression systems could provide precise regulation of ADAR expression to achieve desired editing activity at the optimal time, minimizing potential adverse effects. We anticipate more efficient and safer RNA editing in future studies, leading to breakthroughs in areas such as biosensing and gene therapy. Collectively, our hairpin RNA sensor not only introduces new concepts for ADAR sensor design but also enriches the RNA regulation toolbox with its distinctive features, potentially advancing the development of intelligent sensing technologies.

## Supplementary Material

gkae1146_Supplemental_File

## Data Availability

The data underlying this article will be shared on reasonable request to the corresponding author. Raw reads have been deposited in the Sequence Read Archive under BioProject accession PRJNA1154999.
