# Supplementary material for: Programming ADAR-recruiting hairpin RNA sensor to detect endogenous molecules
Source: Nucleic Acids Res. 2024 Dec 3;53(1):gkae1146. doi: 10.1093/nar/gkae1146 (PMC11724285; doi:10.1093/nar/gkae1146)
Supplement: gkae1146_Supplemental_File [file gkae1146_supplemental_file.docx]

**Programming ADAR-recruiting hairpin RNA sensor to detect endogenous molecules**

Pei-Pei Qin^1^, Pin-Ru Chen^1^, Liu Tan^1^, Xiaohe Chu^1^, Bang-Ce Ye^1,2^, and Bin-Cheng Yin^1,2,3,^*

^1^Institute of Engineering Biology and Health, Collaborative Innovation Center of Yangtze River Delta Region Green Pharmaceuticals, College of Pharmaceutical Sciences, Zhejiang University of Technology, Hangzhou, 310014, China

^2^Lab of Biosystem and Microanalysis, State Key Laboratory of Bioreactor Engineering, Shanghai Collaborative Innovation Center for Biomanufacturing Technology, East China University of Science and Technology, Shanghai 200237, China

^3^School of Chemistry and Chemical Engineering, Shihezi University, Shihezi 832000, Xinjiang, China

Email: binchengyin@ecust.edu.cn

**Supplementary Tables**

**Table S1.** Primer sequences used for plasmid construction.

| Name | Sequence (5’-3’) |
| --- | --- |
| BFP-F | ATAGGGAGAGCCGCCACCATGATGGTGTCTAAGGGCGAAGAGC |
| BFP-R | AAACGGCGCTGCCTCCTCCGCCGCTGCCTCCTCCGCC |
| GFP-F | GCTACTAACTTCAGCCTGCTGAAGCAG |
| GFP-R | CTGATCAGCGGGTTTAAACTTTACTTGTACAGCTCG |
| pCMV-F | AGTTTAAACCCGCTGATCAGCC |
| pCMV-R | GGTGGCGGCTCTCCCTATAG |
| ADAR1^P150^-F | GAGAGCCGCCACCATGAATCCGCGGCAGGGG |
| ADAR1^P150^-R | GGGTTTAAACTTTATACTGGGCAGAGATAAAAGT |
| ADAR1^P110^-F | GAGAGCCGCCACCATGGCCGAGATCAAGGA |
| ADAR1^P110^-R | GGGTTTAAACTTTATACTGGGCAGAGATAAA |
| ADAR2-F | GAGAGCCGCCACCATGGATATAGAAGATGAAG |
| ADAR2-R | GGGTTTAAACTTTCGGGCGTGAGTGAGAACTG |
| ADAR1^P150^(E1008Q)-F | AGGTGGAGAACGGACAAGGCACAATCCCTGTGG |
| ADAR1^P150^(E1008Q)-R | CCACAGGGGATTGTGCCTTGTCCGTTCTCCACCT |
| ADAR2(E488Q)-F | AAAATAGAGTCTGGTCAGGGGACGATTCCA |
| ADAR2(E488Q)-R | TGGAATCGTCCCCTGACCAGACTCTATTTT |
| MCP-F | CATGGCTTCTAACTTTAATCAGTTCG |
| MCP-R | GATGCCGGAGTTTGCTGCG |
| MS2 | ACATGAGGATCACCCATGT |
| NLS | CCAAAGAAGAAGCGGAAAGTC |
| NES | CTGCCTCCACTTGAAAGACTGACACTG |
| Seq-F | ATGTGGACTACAGACTGGAAAGAATCA |
| Seq-R | TTGCCGGTGGTGCAGATGAAC |
| qPCR-actin-F | TAGTTGCGTTACACCCTTTCTTG |
| qPCR-actin-R | TCACCTTCACCGTTCCAGTTT |
| qPCR-GAPDH-F | ACACCCACTCCTCCACCTTTG |
| qPCR-GAPDH-R | TCCACCACCCTGTTGCTGTAG |
| qPCR-TP53-F | TTCCTGAAAACAACGTTCTGTC |
| qPCR-TP53-R | AACCATTGTTCAATATCGTCCG |
| qPCR-HER2-F | CCAGCTCTTTGAGGACAACTAT |
| qPCR-HER2-R | CCAGCTCTTTGAGGACAACTAT |
| qPCR-NF-κB-F | CAGCCTCCAGCCCAGTGAAG |
| qPCR-NF-κB-R | GATGTCTCCACGCCGCTGTC |
| mRNA sensor-NGS-F | AGATACTGCGACCTCCCTAGC |
| mRNA sensor-NGS-R | CCAGGATGGGCACCACC |
| EXO-NGS-F | TTGGCTTTATATATCTTGTGGAAAGGACGA |
| EXO-NGS-R | AGTCCAGCTGTATCCAGTATGTCC |
| GAPDH-NGS-F | CCAGTGGGTGGAATCTCACATTC |
| GAPDH-NGS-R | CAACGAATTTGGCCACAGCAA |
| TP53-NGS-R | CCGAGTGGAAGGAAATTTGCG |
| HER2-NGS-R | GTGCGAGGCACCCAGC |

**Table S2.** Hairpin RNA sequences of reporter plasmids used in the study.

| Name | ^*^Sequence (5’-3’) |
| --- | --- |
| AN^2^C | GCCGUCGAUGUGGGUGGCUCACCCACCUACA**N^2^**CGACGGC |
| N^3^CN^1^ | GCCGUCGAUGUGGGUGGCUCACCCACCUAC**N^3^**C**N^1^**GACGGC |
| a7b9 | GCCGUCGAUGUGGGUGGCUCACCCACCUACACCGACGGC |
| a7b12 | GCCGUCGAUGUGGGUGGAAUAACUCACUAUCCCACCUACACCGACGGC |
| a7b15 | GCCGUCGAUGUGGGUGGAAUAGACUCACUCUAUCCCACCUACACCGACGGC |
| a7b18 | GCCGUCGAUGUGGGUGGAAUAGAUUACUCACUAAUUAUCCCACCUACACCGACGGC |
| a7b21 | GCCGUCGAUGUGGGUGGAAUAGAUUACCACUCACUGGUAAUGUAUCCCACCUACACCGACGGC |
| a10b9 | GCAGCCGUCGAUGUGGGUGGCUCACCCACCUACACCGACGGCUGC |
| a10b12 | GCAGCCGUCGAUGUGGGUGGAAUCUCACAUUCCACCUACACCGACGGCUGC |
| a10b15 | GCAGCCGUCGAUGUGGGUGGAAUAGACUCACUCUAUCCCACCUACACCGACGGCUGC |
| a10b18 | GCAGCCGUCGAUGUGGGUGGAAUAGAUUACUCACUAAUGUAUCCCACCUACACCGACGGCUGC |
| a10b21 | GCAGCCGUCGAUGUGGGUGGAAUAGAUUACCACUCACUGGUAAUGUAUCCCCACUACCACCGACGGCUGC |
| Reporter-1 | GCAGCCGUCGAUGUGGGUGGAAUCUCACAUUCCACCUACACCGACGGCUGC |
| Reporter-2 | GCAGCCGUCGAUGUGGGUGGAAU |
| domain a: 5 nt | GACUCCAGUGGGUGGAAUCUCACAUUCCACCUACUAGAGUCCAGCUGUAUCCAGUAUGUCCAACAAACAGGUUUCA |
| domain a: 3 nt | CUCCAGUGGGUGGAAUCUCACAUUCCACCUACUAGAGUCCAGCUGUAUCCAGUAUGUCCAACAAACAGGUUUCA |
| domain a: 2 nt | UCCAGUGGGUGGAAUCUCACAUUCCACCUACUAGAGUCCAGCUGUAUCCAGUAUGUCCAACAAACAGGUUUCA |
| domain a: 1 nt | CCAGUGGGUGGAAUCUCACAUUCCACCUACUAGAGUCCAGCUGUAUCCAGUAUGUCCAACAAACAGGUUUCA |
| domain a: 0 nt | CAGUGGGUGGAAUCUCACAUUCCACCUACUAGAGUCCAGCUGUAUCCAGUAUGUCCAACAAACAGGUUUCA |
| domain a: PC | AGUGGGUGGAAUCUCACAUUCCACCUACUAGAGUCCAGCUGUAUCCAGUAUGUCCAACAAACAGGUUUCA |
| N^5^ | CCAGUGGGUGGAAUCUCACAUUCCACCUACUAG**N^5^**GUCCAGCUGUAUCCAGUAUGUCCAACAAACAGGUUUCA |
| N^6^CN^7^ | **N^6^**C**N^7^**GUGGGUGGAAUCUCACAUUCCACCUACUAGAGUCCAGCUGUAUCCAGUAUGUCCAACAAACAGGUUUCA |
| *GAPDH* sensor-1 | CCAGUGGGUGGAAUCUCACAUUCCACCUACUAGAUGAGGUCCACCACCCUGUUGCUGUGGCCAAAUUCGUUG |
| *GAPDH* sensor-2 | CCAGUGGGUGGAAUCUCACAUUCCACCUACUAGGUCUUCUGGGUGGCAGUGAUGGCAUGGACUGUGGUCAUG |
| *GAPDH* sensor-3 | CCAGUGGGUGGAAUCUCACAUUCCACCUACUAGCCACCACUGACACGUUGGCAGUGGGGACACGGAAGGCCA |
| *TP53* sensor-1 | CCAGUGGGUGGAAUCUCACAUUCCACCUACUAGUCAUCCAAAUACUCCACACGCAAAUUUCCUUCCACUCGG |
| *TP53* sensor-2 | CCAGUGGGUGGAAUCUCACAUUCCACCUACUAGUCCCCUUUCUUGCGGAGAUUCUCUUCCUCUGUGCGCCGG |
| *TP53* sensor-3 | CCAGUGGGUGGAAUCUCACAUUCCACCUACUAGUACGUGCAAGUCACAGACUUGGCUGUCCCAGAAUGCAAG |
| *HER2* sensor-1 | CCAGUGGGUGGAAUCUCACAUUCCACCUACUAGCAGGGCAUAGUUGUCCUCAAAGAGCUGGGUGCCUCGCAC |
| *HER2* sensor-2 | CCAGUGGGUGGAAUCUCACAUUCCACCUACUAGCCAGACCCAGGUACUCUGGGUUCUCUGCCGUGGGUGUCC |
| *HER2* sensor-3 | CCAGUGGGUGGAAUCUCACAUUCCACCUACUAGGGCCCCAAAAGUCAUCAGCUCCCACACAGUCACACCAUA |
| *HER2* sensor-4 | CCAGUGGGUGGAAUCUCACAUUCCACCUACUAGGACCAGCACGUUCCGAGCGGCCAAGUCCCUGUGUACGAG |
| *HER2* sensor-5 | CCAGUGGGUGGAAUCUCACAUUCCACCUACUAGCAUUCUCCCCAUCAGGGAUCCAGAUGCCCUUGUAGACUG |
| SNV sensor | CCAGUGGGUGGAAUCUCACAUUCCACCUACUAGAGUCCAGCUGUAUCCAGUAUGUCCAACAAACAGGUUUCA |
| ATP sensor | ACGCACCCAGACUGCGCAAGUGCCACAGUUUCUUCCCAACCCUGCGCGGUCUAGGUGCGUGGGUUGGGAAGAAACUGUGGCACUUCGGUGCCAGCAACCC |
| MS2-ATP sensor | ACAUGAGGAUCACCCAUGUACGCACCCAGACUGCGCAAGUGCCACAGUUUCUUCCCAACCCUGCGCGGUCUAGGUGCGUGGGUUGGGAAGAAACUGUGGCACUUCGGUGCCAGCAACCC |
| NF-κB sensor | AACGCACCCAGACUGCGCACUUACAGUUUCAAGUAUCUCCCGGUCUAGGUGCGGGUGAUACUUGAAACUGUAAGGUUGGCGUAUA |
| MS2-NF-κB sensor | ACAUGAGGAUCACCCAUGUAACGCACCCAGACUGCGCACUUACAGUUUCAAGUAUCUCCCGGUCUAGGUGCGGGUGAUACUUGAAACUGUAAGGUUGGCGUAUA |
| control sensor  (random sequence) | AACGCACCCAGACUGCGCAAAGGAUCGGUUCCAGUAUUCCCGGUCUAGGUGCGGGUAUACUGGAACCGCUACUUACACAGGAACAU |

^*^Red characters represent editing site of ADAR2(E488Q). Bolded characters represent the central position of triplet 5’-CN^2^A-3’, the neighboring two bases of 5’-N^1^CN^3^-3’ triplet, the adjacent bases N^5^ on the right side of the triplex 5’-CCA-3’, and the neighboring two bases of 5’-N^6^CN^7^-3’ triplet, respectively. N denotes A, U, C or G. Underlined character represents the MS2 bases sequence.

**Table S3.** siRNA sequences for gene knockdown.

| Name | Sequence (5’-3’) |
| --- | --- |
| *GAPDH*-1 | GUGUGAACCAUGAGAAGUA |
| *GAPDH*-2 | UCAAGAAGGUGGUGAAGCA |
| *GAPDH*-3 | GAAUUUGGCUACAGCAACA |
| *TP53*-1 | GAAAUUUGCGUGUGGAGUA |
| *TP53*-2 | GCACAGAGGAAGAGAAUCU |
| *TP53*-3 | CTGAGGUUGGCUCUGACUG |
| *HER2*-1 | CCCAGAUCUUUGAGGACAA |
| *HER2*-2 | GGUGUGAGAAGUGCAGCAA |
| *HER2*-3 | AAGAGUCCCAACCAUGUCA |
| NF-κB-1 | AAGAAGAAGUGCAGAGGAA |
| NF-κB-2 | CAAAGAAGGACAUGAUAAA |
| NF-κB-3 | CUGGAUGACUCUUGGGAAA |

**Table S4.** Exogenous RNA sequences used in the study.

| Name | ^#^Sequence (5’-3’) |
| --- | --- |
| exogenous RNA= 9 nt | AGUUGGACU |
| exogenous RNA= 18 nt | ACUCCAUACAGUUGGACU |
| exogenous RNA= 27 nt | GUUGGACAUACUCCAUACAGUUGGACU |
| exogenous RNA= 39 nt | UGAAACCUGUUUGUUGGACAUACUGGAUACAGCUGGAC**N^4^** |
| exogenous RNA= 48 nt | AAGGGUCUGUGAAACCUGUUUGUUGGACAUACUGGAUACAGCUGGACU |
| WT RNA | UGAAACCUGUUUGUUGGACAUACUGGAUACAGCUGGACA |
| 26G->C | UGAAACCUGUUUGUUGGACAUACU**C**GAUACAGCUGGACA |
| 27G->C | UGAAACCUGUUUGUUGGACAUACUG**C**AUACAGCUGGACA |
| 28A->C | UGAAACCUGUUUGUUGGACAUACUGG**C**UACAGCUGGACA |
| 29U->C | UGAAACCUGUUUGUUGGACAUACUGGA**C**ACAGCUGGACA |
| 30A->C | UGAAACCUGUUUGUUGGACAUACUGGAU**C**CAGCUGGACA |
| 31C->A | UGAAACCUGUUUGUUGGACAUACUGGAUA**A**AGCUGGACA |
| 32A->C | UGAAACCUGUUUGUUGGACAUACUGGAUAC**C**GCUGGACA |
| 33G->C | UGAAACCUGUUUGUUGGACAUACUGGAUACA**C**CUGGACA |
| 34C->A | UGAAACCUGUUUGUUGGACAUACUGGAUACAG**A**UGGACA |
| 35U->C | UGAAACCUGUUUGUUGGACAUACUGGAUACAGC**C**GGACA |
| 36G->C | UGAAACCUGUUUGUUGGACAUACUGGAUACAGCU**C**GACA |
| 37G->C | UGAAACCUGUUUGUUGGACAUACUGGAUACAGCUG**C**ACA |
| 38A->C | UGAAACCUGUUUGUUGGACAUACUGGAUACAGCUGG**C**CA |
| 39C->A | UGAAACCUGUUUGUUGGACAUACUGGAUACAGCUGGA**A**A |
| mutation | UGAAACCUGUUUGUUGGACAUACUGGAUACAGCUGGAC**U** |
| 30A->* | UGAAACCUGUUUGUUGGACAUACUGGAU*****CAGCUGGACA |
| 33G->* | UGAAACCUGUUUGUUGGACAUACUGGAUACA*****CUGGACA |
| 36G->* | UGAAACCUGUUUGUUGGACAUACUGGAUACAGCU*****GACA |
| 38A->* | UGAAACCUGUUUGUUGGACAUACUGGAUACAGCUGG*****CA |
| 39C->* | UGAAACCUGUUUGUUGGACAUACUGGAUACAGCUGGA*****A |
| 40A->* | UGAAACCUGUUUGUUGGACAUACUGGAUACAGCUGGAC***** |
| 30-->C | UGAAACCUGUUUGUUGGACAUACUGGAUA**C**CAGCUGGACA |
| 33-->C | UGAAACCUGUUUGUUGGACAUACUGGAUACAG**C**CUGGACA |
| 36-->C | UGAAACCUGUUUGUUGGACAUACUGGAUACAGCUG**C**GACA |
| 38-->C | UGAAACCUGUUUGUUGGACAUACUGGAUACAGCUGGA**C**CA |
| 39-->G | UGAAACCUGUUUGUUGGACAUACUGGAUACAGCUGGAC**G**A |

**^#^**Orange sequences represent the exogenous RNA sequence of *NRAS*. Gray, blue, and green sequences represent single base substitutions, deletions, and insertions, respectively.

**Table S5.** The information of RNA aptamers used in this work.

| Target | Sequence (5’-3’) | K_d_ | Reference |
| --- | --- | --- | --- |
| ATP | GGGUUGGGAAGAAACUGUGGCACUUCGGUGCCAGCAACCC | 6-8 μM | (1) |
| NF-κB | GAUACUUGAAACUGUAAGGUUGGCGUAUA | 5 nM | (2,3) |

**Supplementary Figures**

**
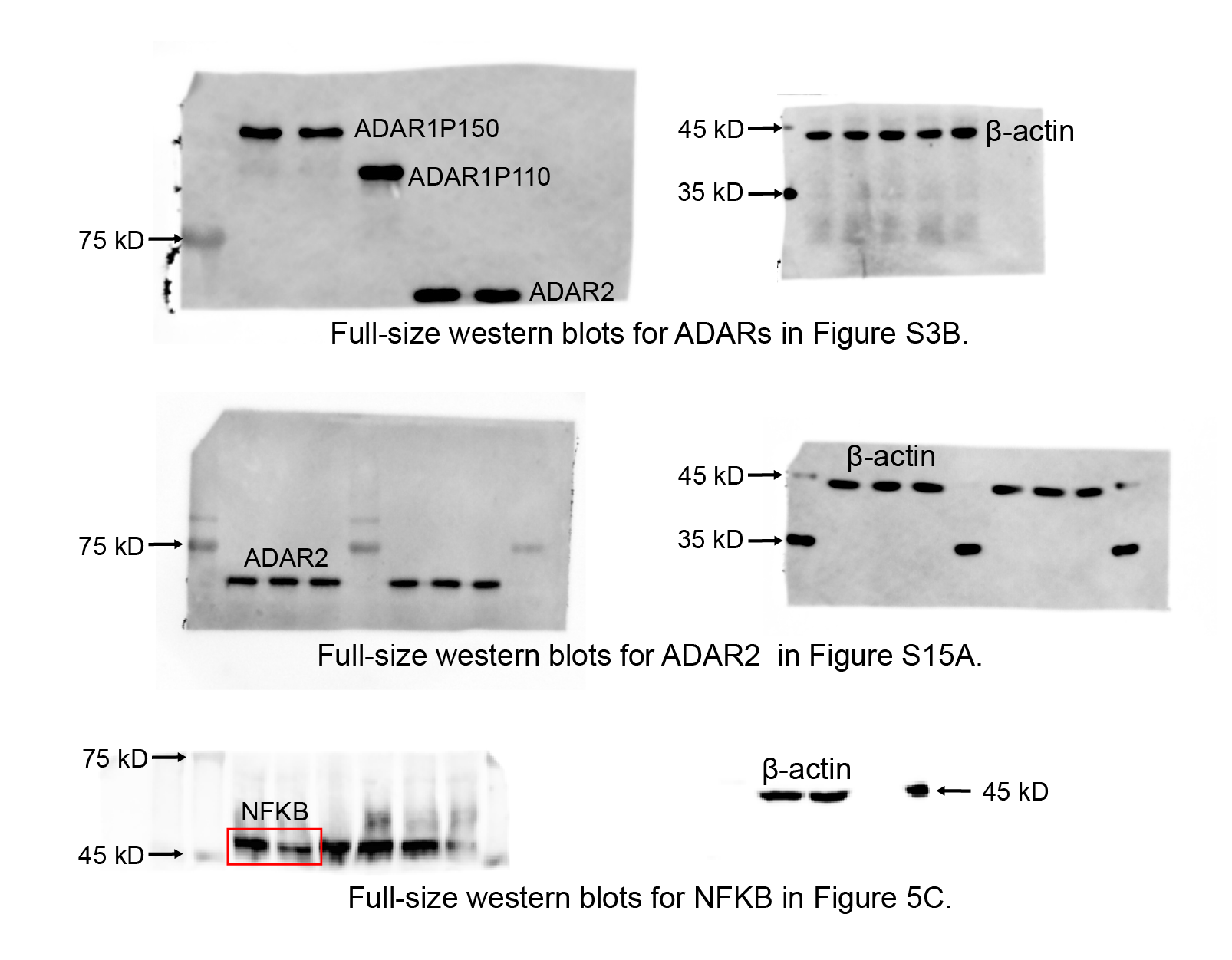
**

**Figure S1.** Full-size blots with molecular weight markers for all prepared proteins in the work.


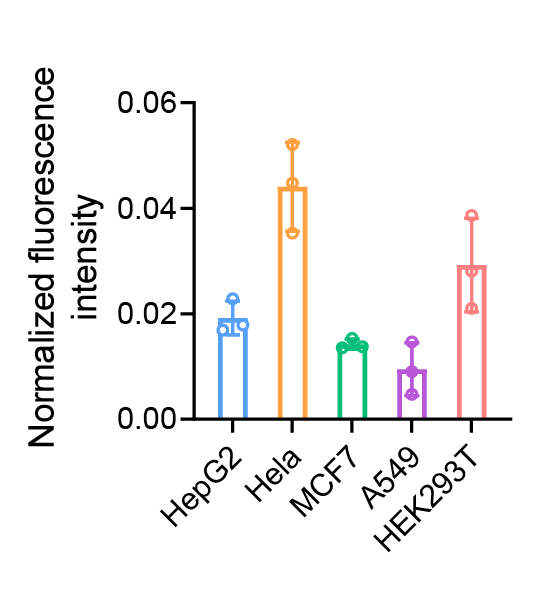


**Figure S2.** Sensing effect analysis of endogenous ADAR. Hairpin sensor efficiency of endogenous ADAR proteins in five cell lines (HepG2, HeLa, MCF7, A549, and HEK293T cells). Values are mean ± s.e.m. with n=3 from three independent experiments.


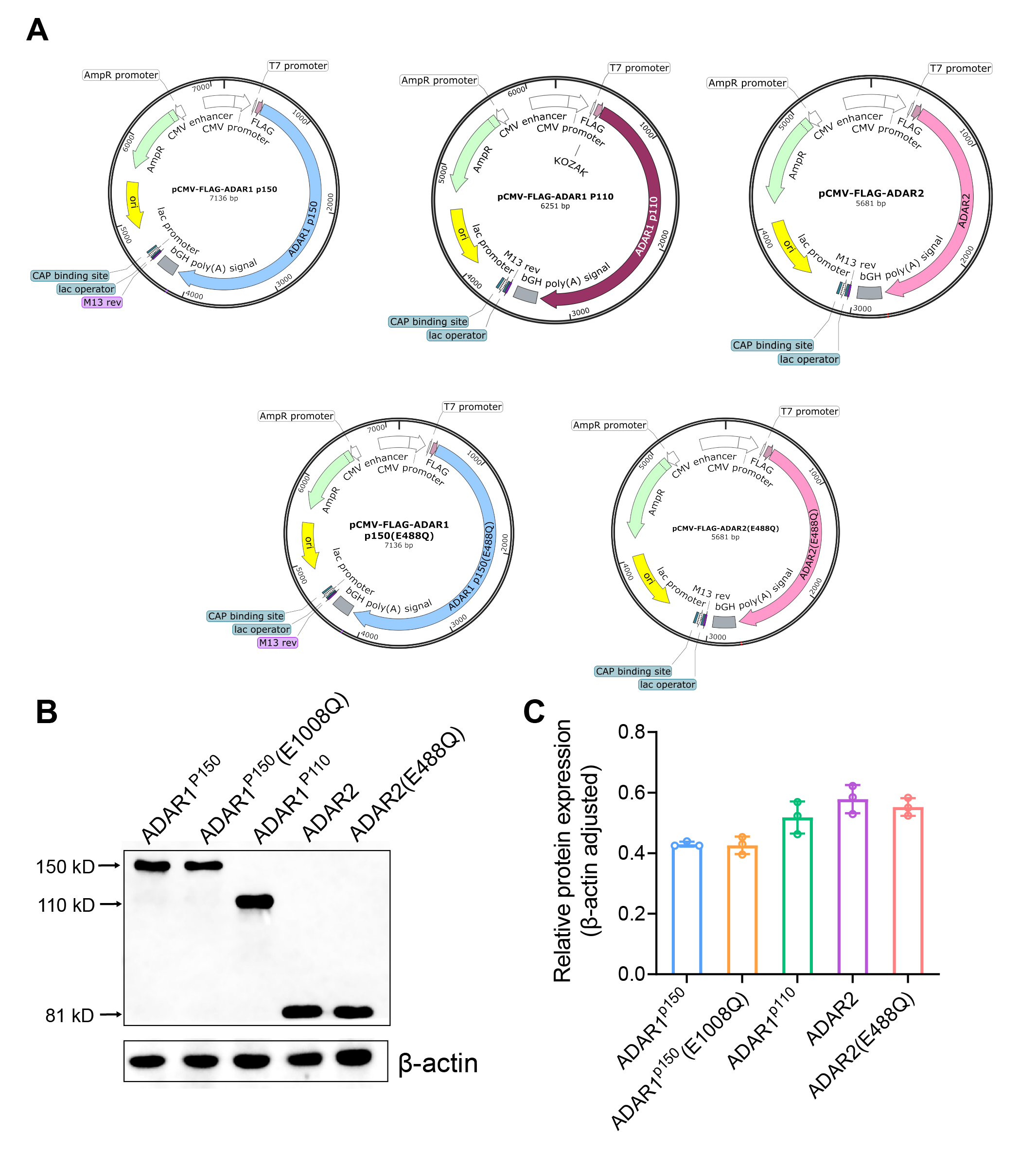


**Figure S3.** (**A**) Maps of five ADAR protein plasmids expressing ADAR1^P150^, ADAR1^P110^, ADAR2, ADAR1^P150^(E1008Q), and ADAR2(E488Q). (**B**) Validation of ADAR protein expression by western blotting. (**C**) Quantitation analysis of ADAR protein expression using β-actin as a control. One-way ANOVA was used for comparison of more than two groups. ***p* < 0.01. Values are mean ± s.e.m. with n=3 from three independent experiments.

**
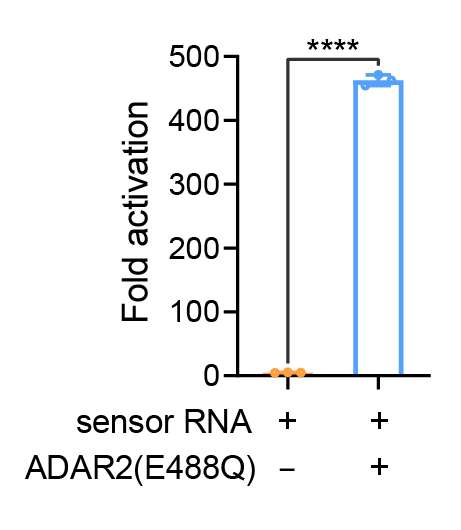
**

**Figure S4.** Effect of exogenous ADAR2(E488Q) addition on sensing effectiveness. One-way ANOVA was used for comparison of more than two groups. *****p* < 0.0001. Values are mean ± s.e.m. with n=3 from three independent experiments.


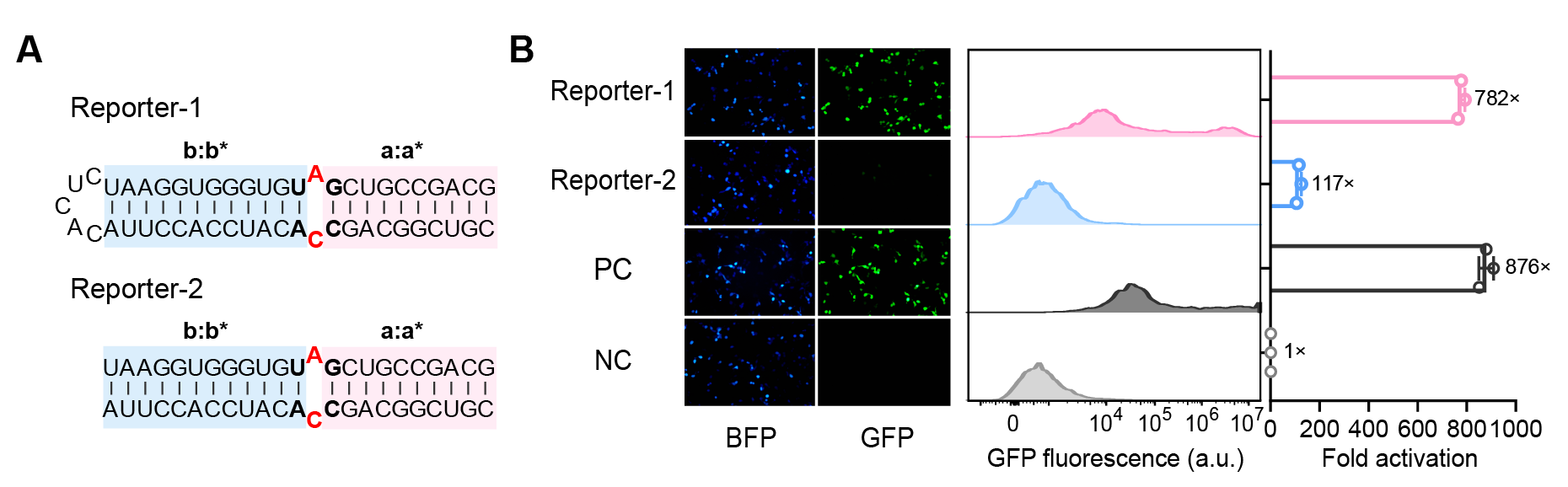


**Figure S5.** Effect of dsRNA structures on hairpin sensor efficiency. (**A**) Schematic of structures of Reporter-1 with an intramolecular duplex and Reporter-2 with an intermolecular duplex. (**B**) Left, fluorescence images of Reporter-1 and Reporter-2 acting with ADAR2(E488Q), respectively; Middle, flow cytometry analysis of the effect of Reporter-1 and Reporter-2 on ADAR2(E488Q); Right, fold activation calculated based on flow cytometry data. PC, positive control with a hairpin RNA replacing UAG by UGG (tryptophan); NC, negative control with a reporter plasmid containing a UAG-bearing hairpin RNA but without the addition of ADAR plasmid. Values are mean ± s.e.m. with n=3 from three independent experiments.


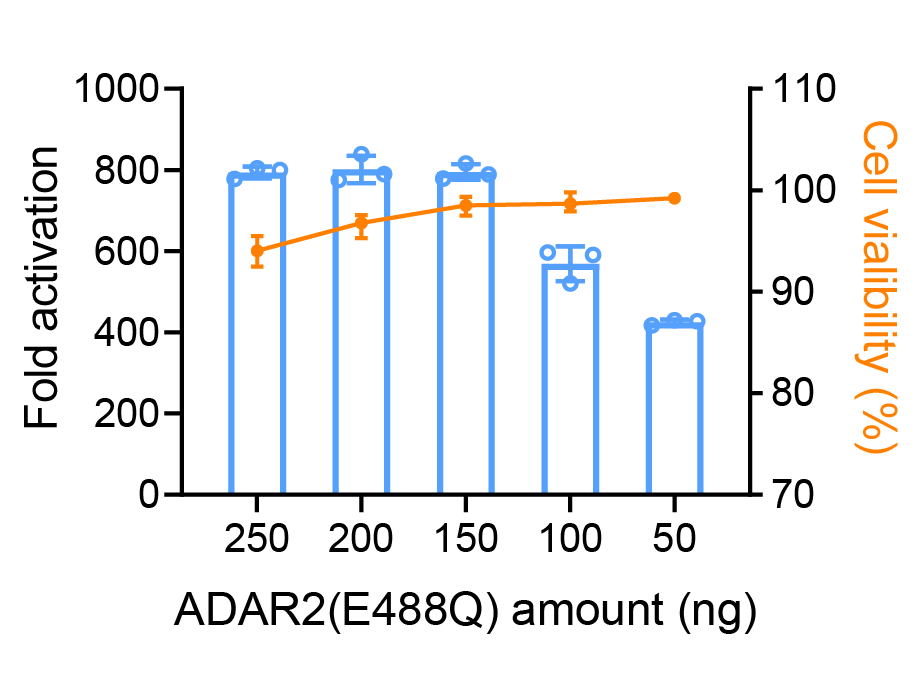


**Figure S6.** Optimization of transfection concentration of ADAR2(E488Q) plasmids in HEK293T cells. Fold activation treated with varying amounts of ADAR2(E488Q) plasmids (50, 100, 150, 200, and 250 ng). Effect of transfection amounts of ADAR2(E488Q) plasmids on HEK239T cell viability tested by CCK-8 kit. Values are mean ± s.e.m. with n=3 from three independent experiments.


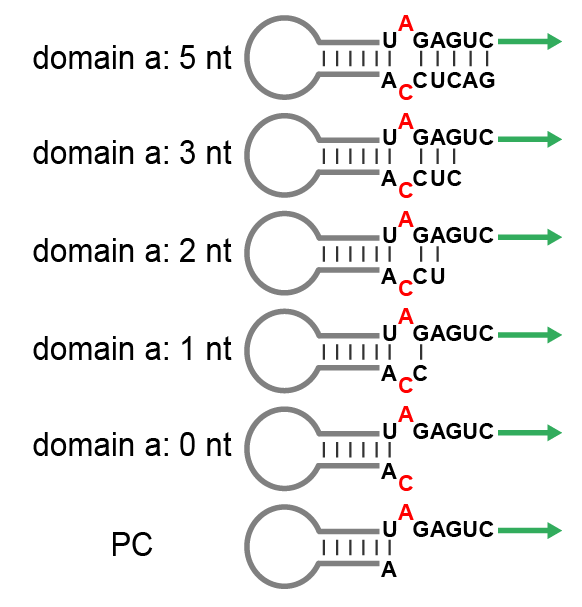


**Figure S7.** Schematic of domain **a** with length ranging from 5 to 0 nt. PC, positive control with a hairpin RNA deleting domain **a** and a neighboring cytosine.


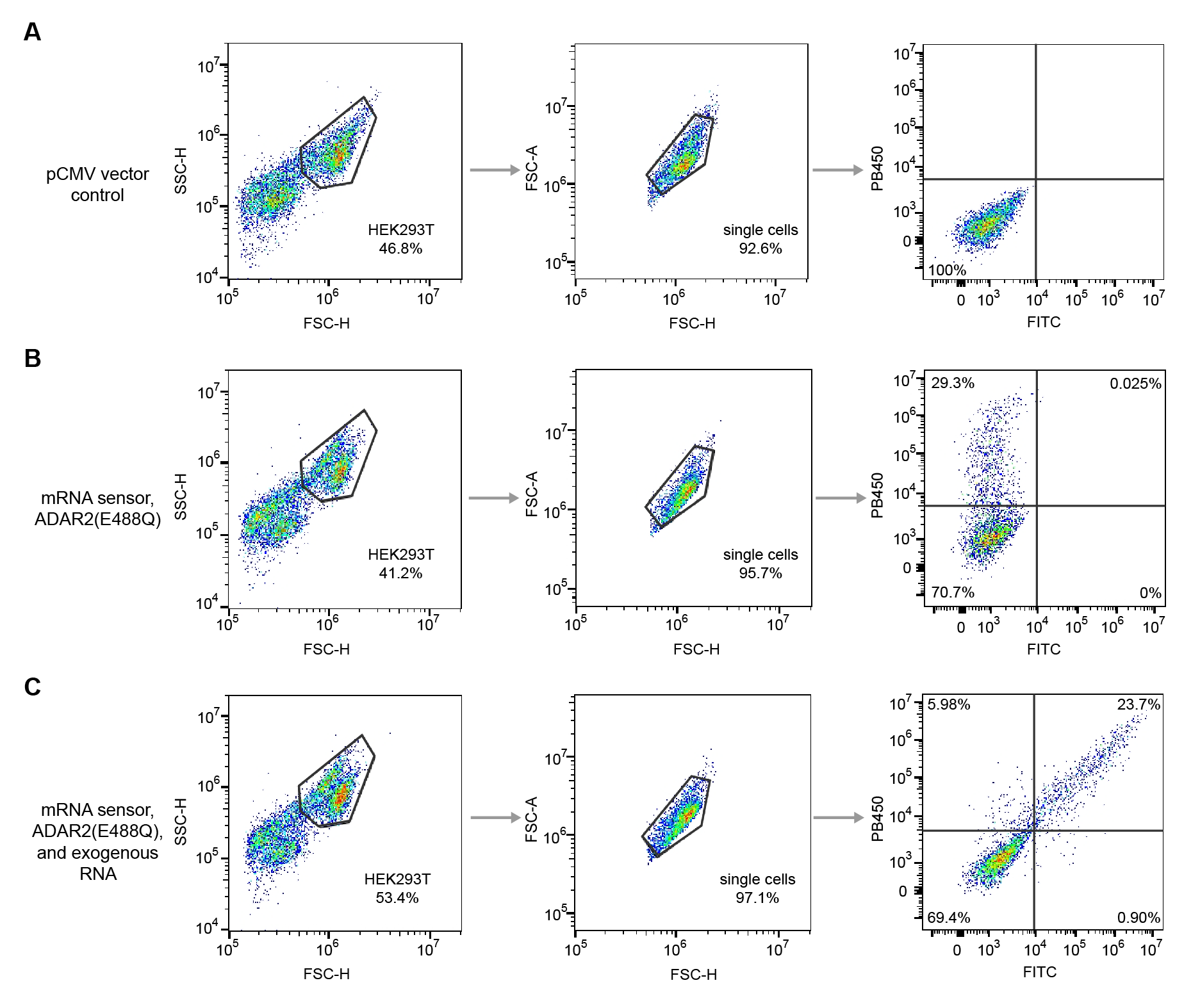


**Figure S8.** Gating strategy for flow cytometry analysis. (**A**) Gating was drawn using control transfected with the pCMV vector plasmid. (**B**) Gates overlaid on HEK293T cell population transfected with mRNA sensor and ADAR2(E488Q) plasmid. (**C**) Gates overlaid on HEK293T cell population transfected with mRNA sensor, exogenous RNA, and ADAR2(E488Q) plasmid.


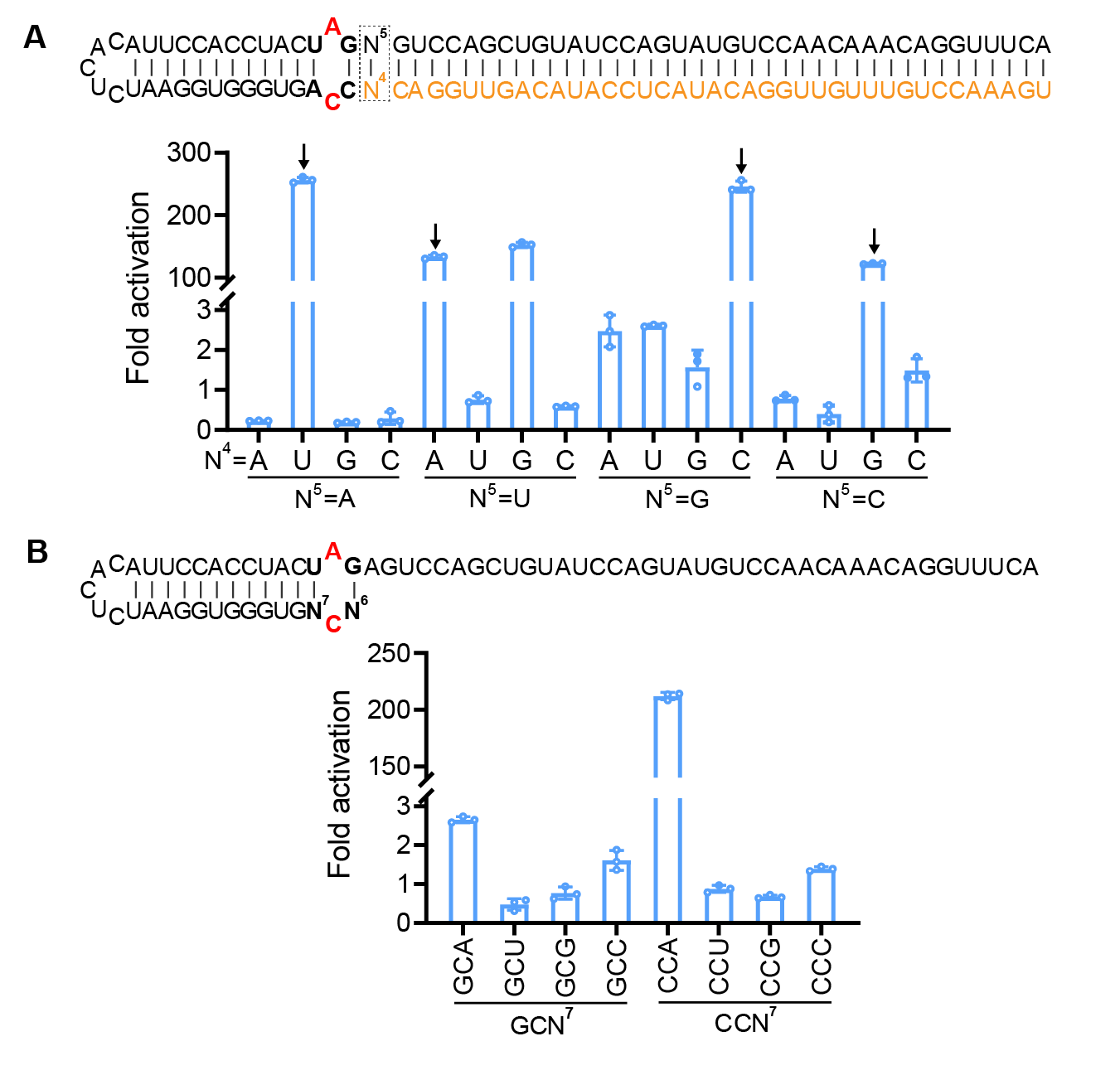


**Figure S9.** (**A**) Effects of base stacking effects arising from base combinations N^4^:N^5^ adjacent to the CG closure of editing loop. N=A, U, G, or C. (**B**) Effect of UAG anticodon 5’-N^6^CN^7^-3’ triplet base combinations on the sensing effect of mRNA sensor (N^6^=G, C). Values are mean ± s.e.m. with n=3 from three independent experiments.


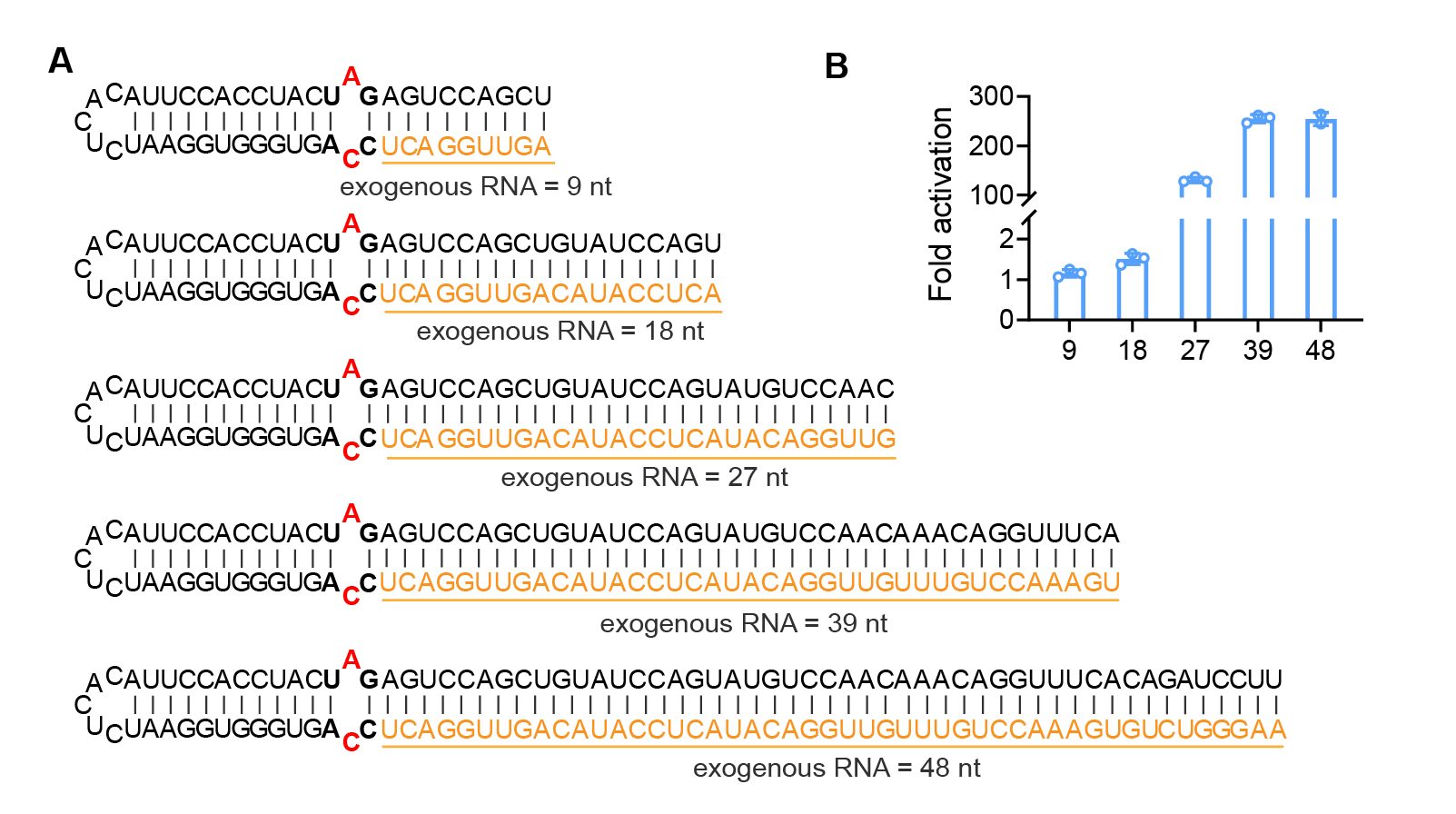


**Figure S10.** Exploration of the length of mRNA sensor binding to targets. (**A**) Schematic of exogenous RNAs with lengths of 9, 18, 27, 39 and 48 nt binding to the mRNA sensor. (**B**) Effect of exogenous RNAs of different lengths on sensor binding. Values are mean ± s.e.m. with n=3 from three independent experiments.


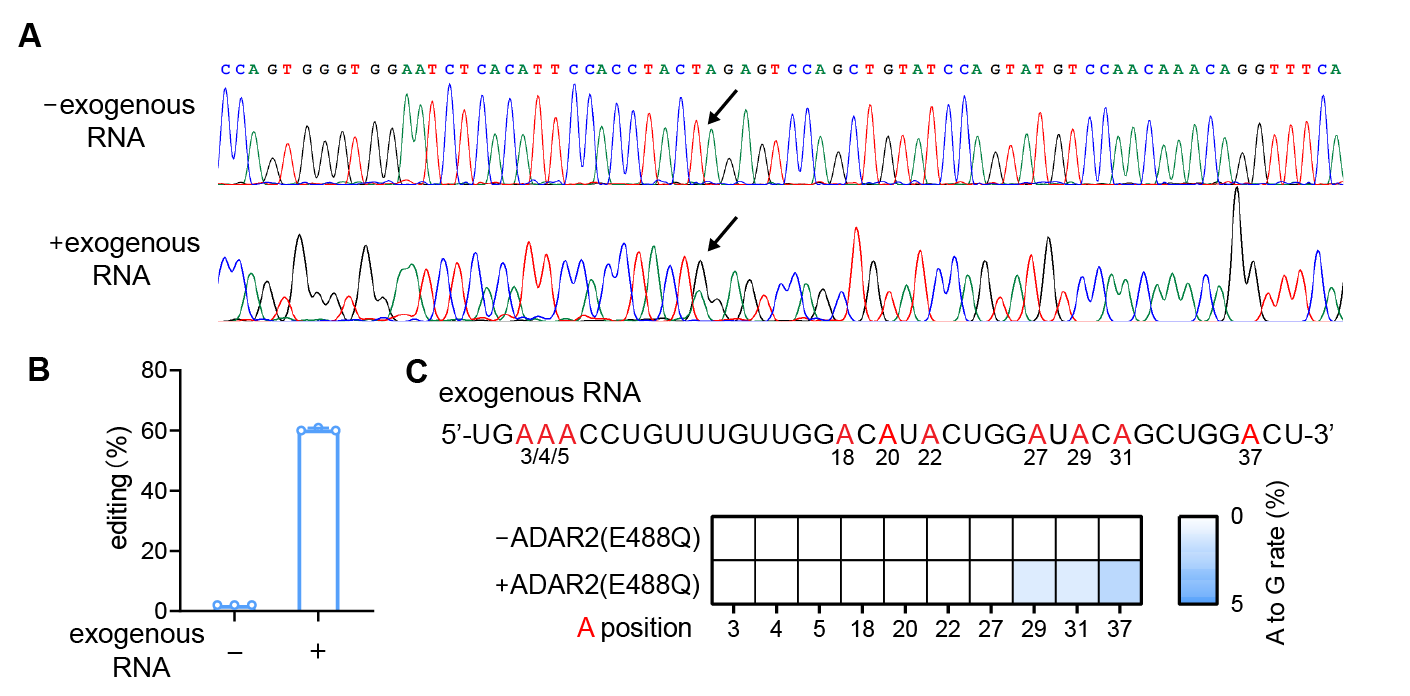


**Figure S11.** Sanger sequencing results (**A**) and quantification results (**B**) for hairpin sensors in the presence or absence of exogenous RNA. (**C**) Bystander editing levels of sensors hybridized with exogenous RNA. Heatmaps depict the conversion of A to I(G). Values are mean ± s.e.m. with n=3 from three independent experiments.


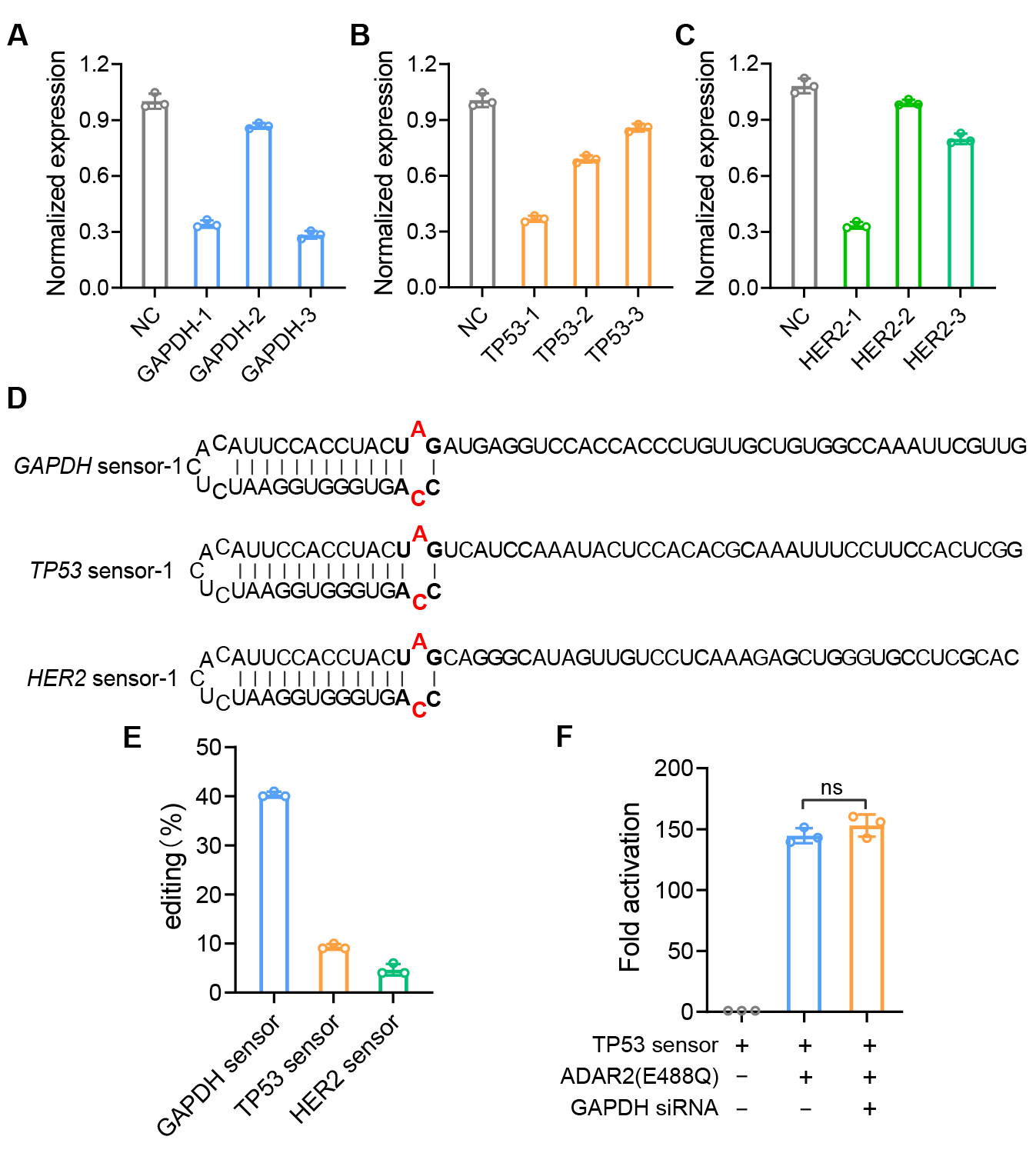


**Figure S12.** Screening of siRNAs for targeted knockdown of (**A**) *GAPDH* mRNA, (**B**) *TP53* mRNA, and (**C**) *HER2* mRNA in HEK293T cells. For each target mRNA, we tested three siRNAs. NC, negative control using siRNA with a random sequence. (**D**) Detailed hairpin RNA sequences for *GAPDH* sensor, *TP53* sensor, and *HER2* sensor. (**E**) Quantification of the A-to-I editing rate for the *GAPDH*, *TP53*, and *HER2* sensors using NGS. (**F**) Specificity investigation of hairpin RNA sensor to detect *TP53* mRNA in the presence of siRNA targeting *GAPDH* mRNA. NC is a positive control without siRNA addition. One-way ANOVA was used for comparison of more than two groups. ns, not significant. Values are mean ± s.e.m. with n=3 from three independent experiments.


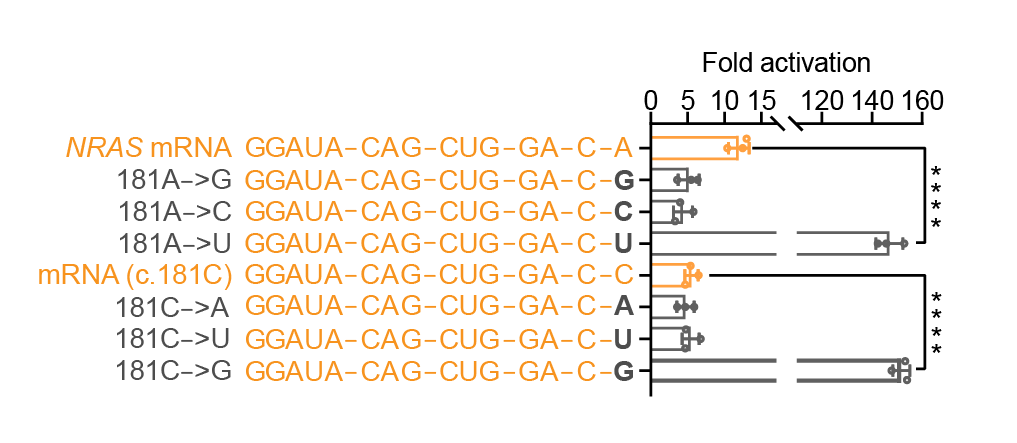


**Figure S13.** Detection of A-U and C-G complementary and their mutations by measuring GFP fluorescence. One-way ANOVA was used for comparison of more than two groups. *****p* < 0.0001. Values are mean ± s.e.m. with n=3 from three independent experiments.

**
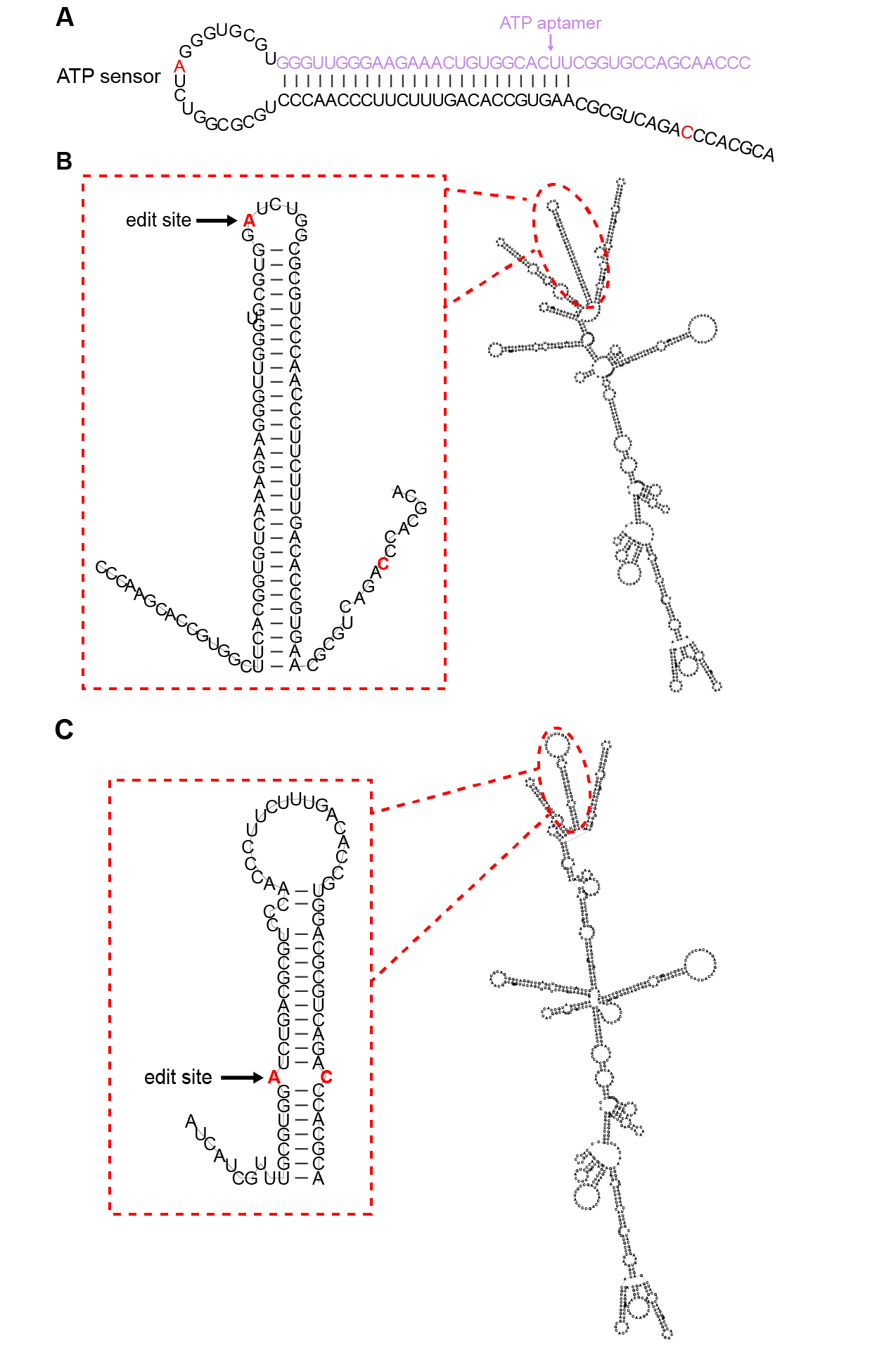
**

**Figure S14.** (**A**) Detailed hairpin RNA sequences for ATP sensor. (**B**) Secondary structure prediction of ATP sensor and translation products with ATP aptamer using the [RNAfold](http://rna.tbi.univie.ac.at/cgi-bin/RNAWebSuite/RNAfold.cgi) web server. (**C**) Secondary structure prediction of ATP sensor and translation products without ATP aptamer using the RNAfold web server.


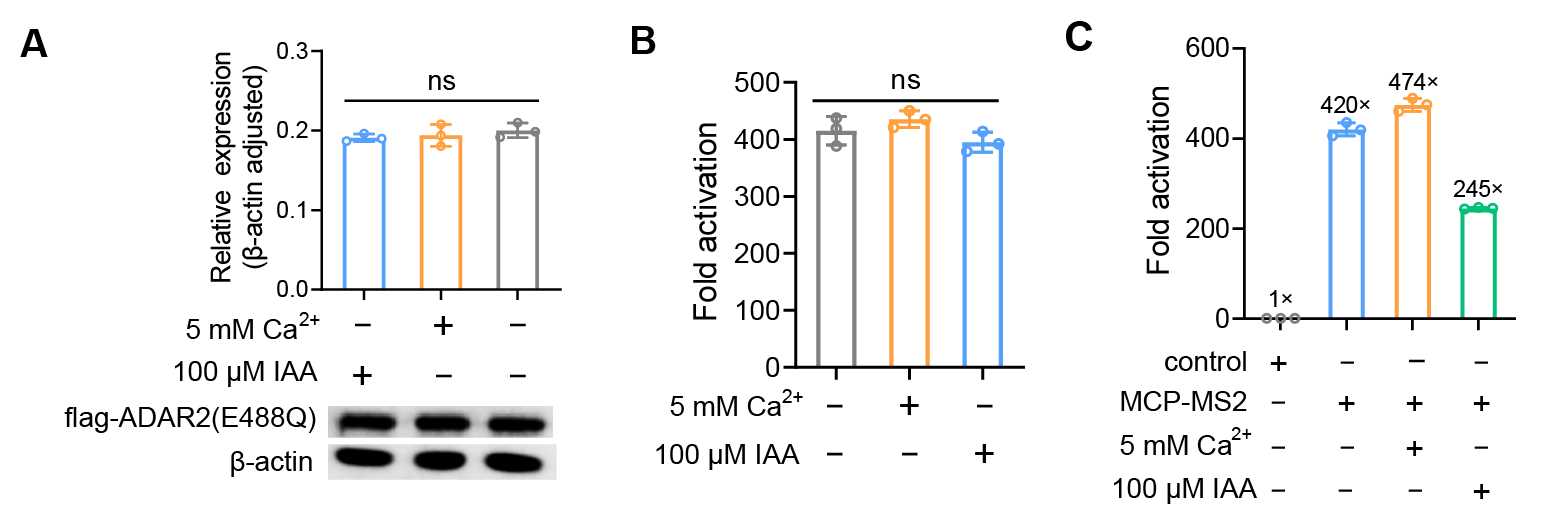


**Figure S15.** (**A**) Effects of exogenous stimulation with IAA and Ca^2+^ on the ADAR2(E488Q) expression by western blotting and quantitation. (**B**) Effects of exogenous stimulation with IAA and Ca^2+^ on the function of hairpin RNA sensors with structural domains **a:a*** and **b:b*** of 7 bp and 9 bp. (**C**) Effects of exogenous stimulation with IAA and Ca^2+^ sensing performance of ATP sensor coupled with the MCP-MS2 system. Control group used random sequences instead of ATP aptamer sequences. One-way ANOVA was used for comparison of more than two groups. ns, not significant. Values are mean ± s.e.m. with n=3 from three independent experiments.


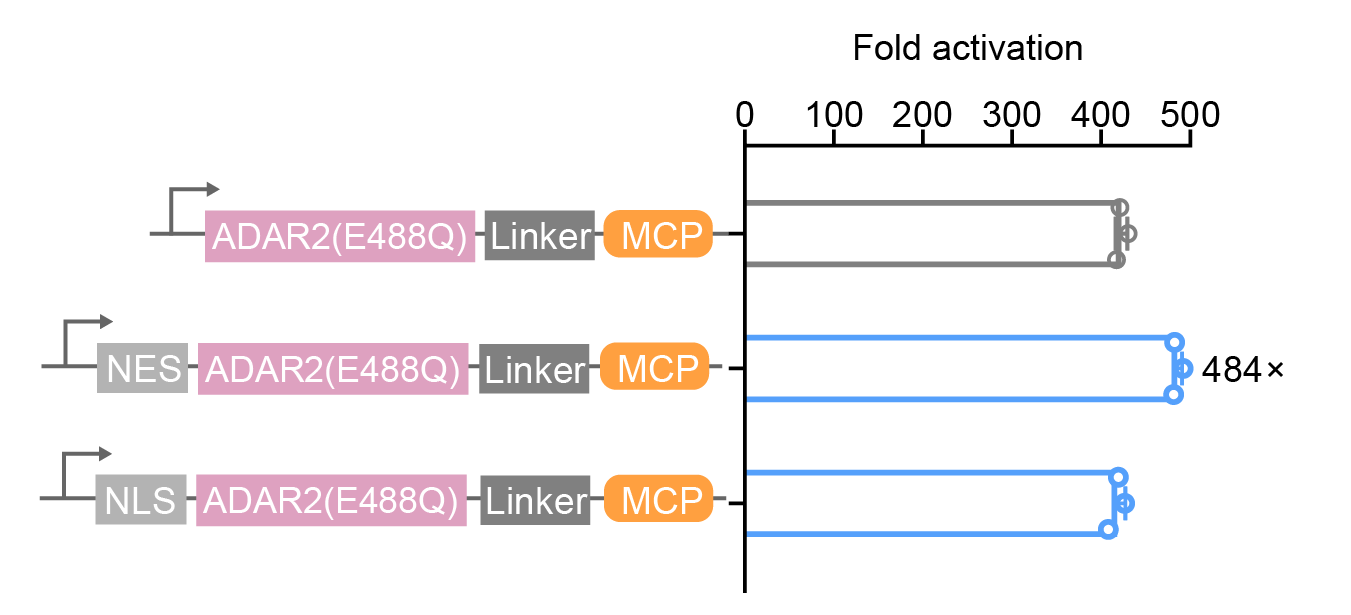


**Figure S16.** Effect of nuclear localization sequence (NLS) and nuclear export sequence (NES) on ATP sensor performance. One-way ANOVA was used for comparison of more than two groups. Values are mean ± s.e.m. with n=3 from three independent experiments.


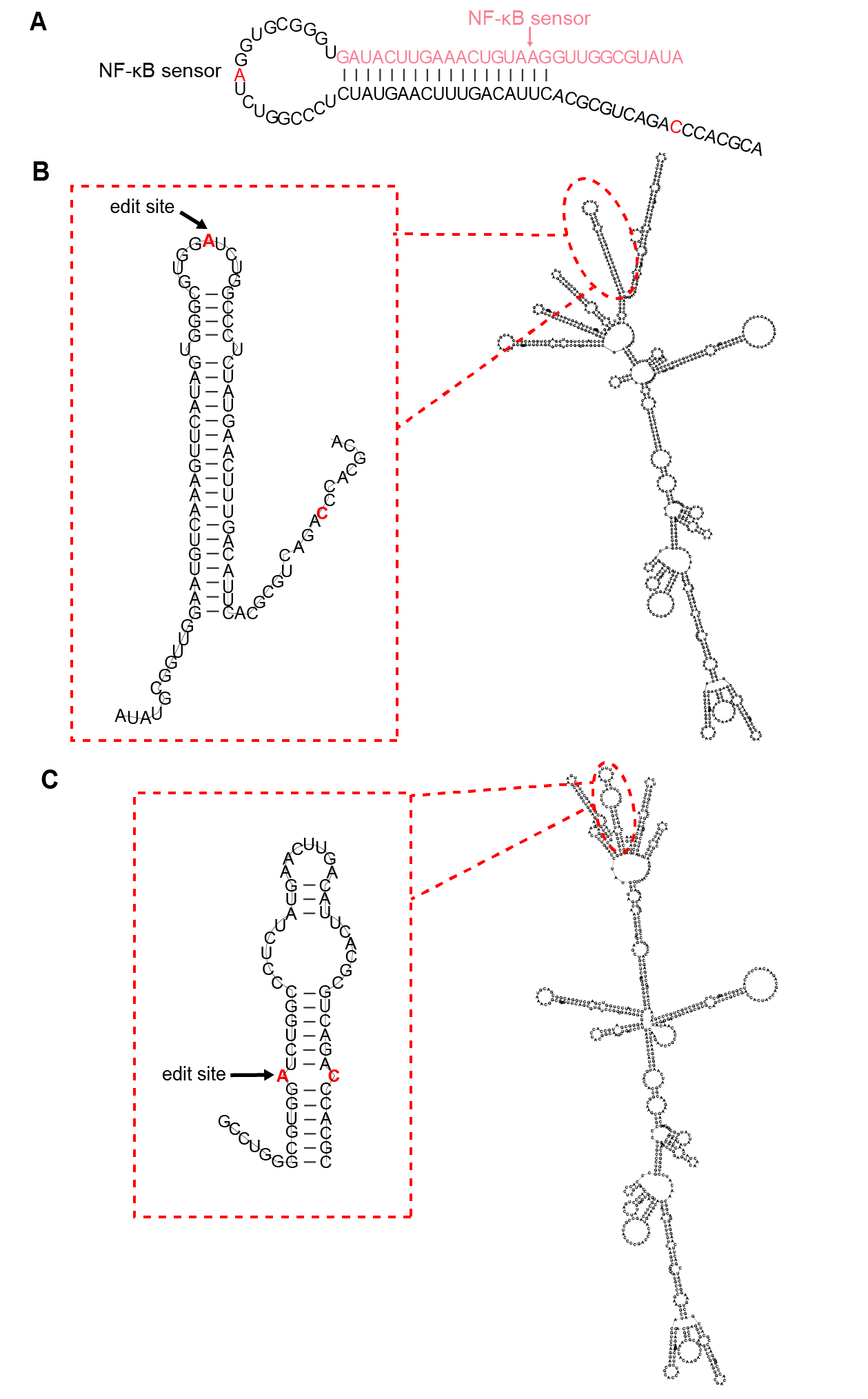


**Figure S17.** (**A**) Detailed hairpin RNA sequences for NF-κB sensor. (**B**) Secondary structure prediction of NF-κB sensor and translation products with NF-κB aptamer using the RNAfold web server. (**C**) Secondary structure prediction of NF-κB sensor and translation products without NF-κB aptamer using the RNAfold web server.


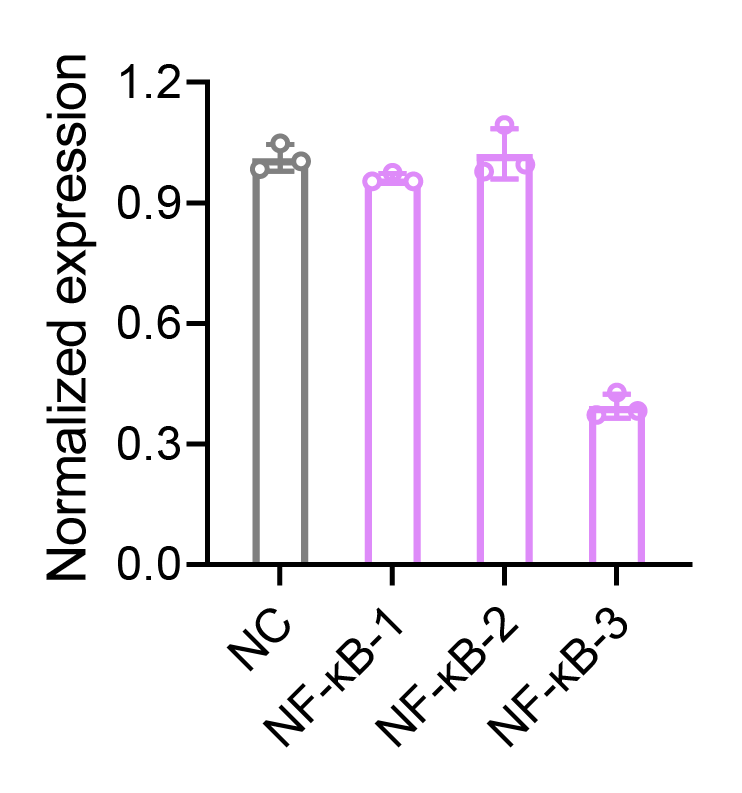


**Figure S18.** siRNA screening for *NF-κB* mRNA knockdown. NC, negative control using siRNA with a random sequence. Values are mean ± s.e.m. with n=3 from three independent experiments.


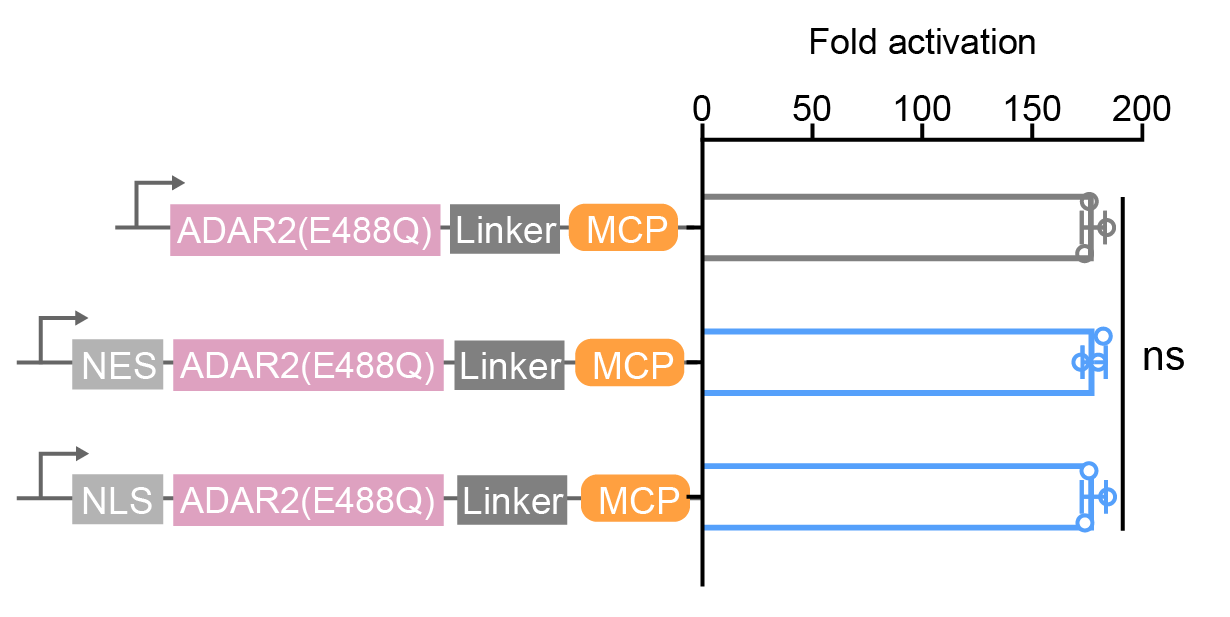


**Figure S19.** Effect of nuclear localization sequence (NLS) and nuclear export sequence (NES) on NF-κB sensor performance. One-way ANOVA was used for comparison of more than two groups. ns, not significant. Values are mean ± s.e.m. with n=3 from three independent experiments.

**REFERENCES**

1. Mandana,S. and Szastak,J.W. (1993) An RNA motif that binds ATP. *Nature*, **364**, 550–553.

2. Reiter,N.J., Maher,L.J. and Butcher,S.E. (2008) DNA mimicry by a high-affinity anti-NF-κB RNA aptamer. *Nucleic Acids Res.*, **36**, 1227-1236.

3. Gelinas,A.D., Davies,D.R. and Janjic,N. (2016) Embracing proteins: structural themes in aptamer–protein complexes. *Curr. Opin. Struct. Biol.*, **36**, 122-132.
